# Genome-Wide Variation, Candidate Regions and Genes Associated With Fat Deposition and Tail Morphology in Ethiopian Indigenous Sheep

**DOI:** 10.3389/fgene.2018.00699

**Published:** 2019-01-09

**Authors:** Abulgasim Ahbara, Hussain Bahbahani, Faisal Almathen, Mohammed Al Abri, Mukhtar Omar Agoub, Ayelle Abeba, Adebabay Kebede, Hassan Hussein Musa, Salvatore Mastrangelo, Fabio Pilla, Elena Ciani, Olivier Hanotte, Joram M. Mwacharo

**Affiliations:** ^1^School of Life Sciences, University of Nottingham, Nottingham, United Kingdom; ^2^Department of Zoology, Faculty of Sciences, Misurata University, Misurata, Libya; ^3^Department of Biological Sciences, Faculty of Science, Kuwait University, Safat, Kuwait; ^4^Department of Veterinary Public Health and Animal Husbandry, College of Veterinary Medicine, King Faisal University, Al-Ahsa, Saudi Arabia; ^5^Department of Animal and Veterinary Sciences, College of Agriculture and Marine Sciences, Sultan Qaboos University, Muscat, Oman; ^6^Agricultural Research Center, Misurata, Libya; ^7^Debre Berhan Research Centre, Debre Berhan, Ethiopia; ^8^Amhara Regional Agricultural Research Institute, Bahir Dar, Ethiopia; ^9^LiveGene, International Livestock Research Institute, Addis Ababa, Ethiopia; ^10^Faculty of Medical Laboratory Sciences, University of Khartoum, Khartoum, Sudan; ^11^Dipartimento di Scienze Agrarie e Forestali, Viale delle Scienze, Università Palermo, Palermo, Italy; ^12^Dipartimento Agricoltura, Ambiente e Alimenti, Università degli Studi del Molise, Campobasso, Italy; ^13^Dipartimento di Bioscienze, Biotecnologie e Biofarmaceutica, Università degli Studi di Bari “Aldo Moro ”, Bari, Italy; ^14^Small Ruminant Genomics, International Center for Agricultural Research in the Dry Areas (ICARDA), Addis Ababa, Ethiopia

**Keywords:** admixture, Africa, fat-tail, *Ovis aries*, thin-tail

## Abstract

Variations in body weight and in the distribution of body fat are associated with feed availability, thermoregulation, and energy reserve. Ethiopia is characterized by distinct agro-ecological and human ethnic farmer diversity of ancient origin, which have impacted on the variation of its indigenous livestock. Here, we investigate autosomal genome-wide profiles of 11 Ethiopian indigenous sheep populations using the Illumina Ovine 50 K SNP BeadChip assay. Sheep from the Caribbean, Europe, Middle East, China, and western, northern and southern Africa were included to address globally, the genetic variation and history of Ethiopian populations. Population relationship and structure analysis separated Ethiopian indigenous fat-tail sheep from their North African and Middle Eastern counterparts. It indicates two main genetic backgrounds and supports two distinct genetic histories for African fat-tail sheep. Within Ethiopian sheep, our results show that the short fat-tail sheep do not represent a monophyletic group. Four genetic backgrounds are present in Ethiopian indigenous sheep but at different proportions among the fat-rump and the long fat-tail sheep from western and southern Ethiopia. The Ethiopian fat-rump sheep share a genetic background with Sudanese thin-tail sheep. Genome-wide selection signature analysis identified eight putative candidate regions spanning genes influencing growth traits and fat deposition (*NPR2, HINT2, SPAG8, INSR*), development of limbs and skeleton, and tail formation (*ALX4, HOXB13, BMP4*), embryonic development of tendons, bones and cartilages (*EYA2, SULF2*), regulation of body temperature (*TRPM8*), body weight and height variation (*DIS3L2*), control of lipogenesis and intracellular transport of long-chain fatty acids (*FABP3*), the occurrence and morphology of horns (*RXFP2*), and response to heat stress (*DNAJC18)*. Our findings suggest that Ethiopian fat-tail sheep represent a uniquely admixed but distinct genepool that presents an important resource for understanding the genetic control of skeletal growth, fat metabolism and associated physiological processes.

## Introduction

African indigenous sheep originated in the Near East. They arrived, in the first instance, in North Africa *via* the Isthmus of Suez by the seventh millennium before present (BP) (Marshall, 2000). These sheep were of thin-tail type and their dispersion southwards into East Africa followed possibly the Nile river valley and the Red Sea coastline (Blench and MacDonald, 2006; Gifford-Gonzalez and Hanotte, 2011). The second wave brought fat-tail sheep into North and Northeast Africa *via* two entry points, the Isthmus of Suez and the Horn of Africa across the straits of Bab-el-Mandeb, respectively. The fat-rump sheep are a recent introduction and represent the third wave of arrival and dispersal of the species into eastern Africa (Epstein, 1971; Ryder, 1983; Marshall, 2000).

Sheep fulfill important socio-cultural and economic roles in the Horn of Africa. In Ethiopia they provide a wide range of products, including meat, milk, skin, hair, and manure, and are a form of savings and investment (Assefa et al., 2015). Ethiopia hosts many indigenous breeds of sheep, with currently 14 recognized populations/breeds, which are defined based on their geographic location and/or the ethnic communities managing them (Gizaw, 2008). Based on structure analysis, Edea et al. (2017) showed that the five Ethiopian indigenous sheep populations they analyzed clustered together based on their geographic distribution and tail phenotypes.

Fat depots act as an energy reserve that allows sheep to survive extreme environments and conditions such as prolonged droughts, cold, and food scarcity (Atti et al., 2004; Nejati-Javaremi et al., 2007; Moradi et al., 2012). Based on the combination of tail type and length, Ethiopian indigenous sheep can be classified as short fat-tail, long fat-tail, thin-tail, and fat-rump sheep. The short fat-tail inhabit sub-alpine mountainous regions, the long fat-tail predominate in mid- to high-altitude environments and the fat-rump sheep occur in semi-arid and arid environments (Gizaw et al., 2007). These populations are considered to be adapted to their production environments and they represent an important model species for investigating and enhancing our knowledge on the genome profiles of environmental adaptation, tail morphology, and fat localization.

Different approaches, that contrast groups of fat- and thin-tail sheep, have been used to identify candidate regions and genes associated with tail formation and morphotypes. Moradi et al. (2012) identified three regions on chromosomes 5, 7 and X associated with tail fat deposition in Iranian breeds. Using two fat-tail (Laticauda and Cyprus fat-tail) and 13 Italian thin-tail breeds, Moioli et al. (2015) identified *BMP2* and *VRTN* as the likely candidate genes explaining fat-tail phenotype in the studied populations/breeds. Zhu et al. (2016) detected several copy number variations intersecting genes (*PPARA, RXRA*, and *KLF11*) associated with fat deposition in three Chinese native sheep (Large-tail Han, Altay, and Tibetan). Several candidate genes with possible links to fat-tail development, i.e., *HOXA11, BMP2, PPP1CC, SP3, SP9, WDR92, PROKR1*, and *ETAA1*, were identified using genome scans that contrasted fat- and thin-tail Chinese sheep (Yuan et al., 2017). Whole genome sequencing of extremely short-tail Chinese sheep revealed the *T* gene as the best possible candidate, among other nine genes, influencing tail size, following its association with vertebral development (Zhi et al., 2018). There is, so far, no information on the genetic basis of variation in tail fat distribution and size in African indigenous sheep.

In this study, using the Ovine 50 K SNP BeadChip genotypes, we investigated the (i) genetic relationships and structure within and between Ethiopian indigenous sheep of different fat-tail morphotypes alongside other sheep populations and breeds from the Caribbean, European, Middle East, China and Africa, and (ii) candidate genome regions and genes associated with tail morphology, fat deposition and possible eco-climatic adaptation in African indigenous sheep. For the latter, 11 Ethiopian indigenous sheep of different fat-tail morphotypes and two populations of thin-tail sheep from Sudan were analyzed.

## Materials and Methods

### DNA Samples and SNP Genotyping

**Figure 1:**
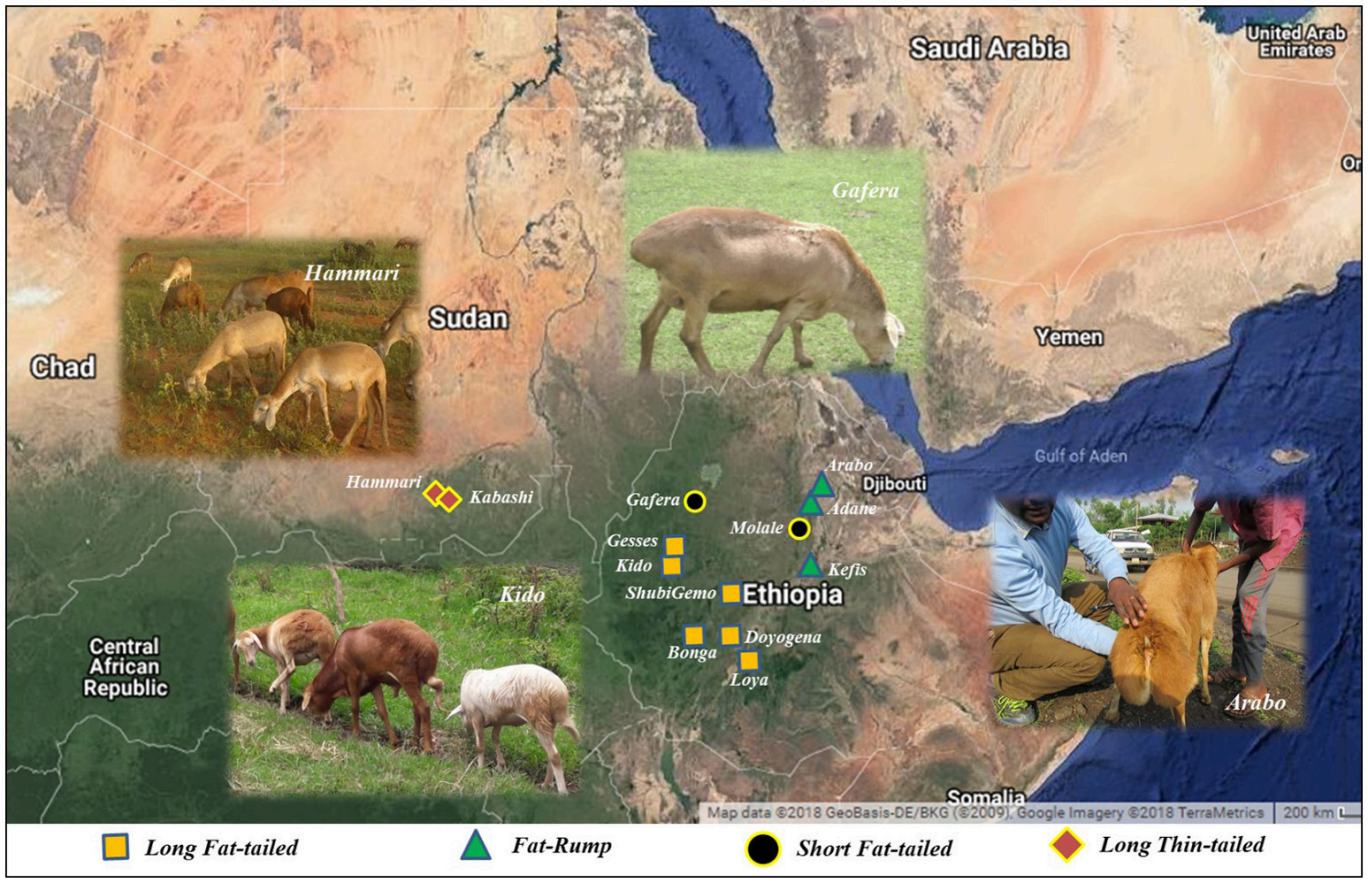
The locations where the Ethiopian and Sudanese sheep populations used in this study were sampled.

The sampling strategy targeted breeds of indigenous sheep from different geographic regions in Ethiopia (Table 1 and Figure 1). Geographic positioning system (GPS) coordinates were recorded for all the populations. We used altitude to determine the agro-eco-climatic zones of the geographic locations where the sheep were sampled. All efforts were made to include populations representing the different tail phenotypes found in Ethiopia. Twenty DNA samples from two thin-tail sheep, Hammari and Kabashi, were obtained from Sudan. Genomic DNA was extracted from 146 ear tissue samples, collected from 11 Ethiopian indigenous sheep populations, using the NucleoSpin® Tissue Kit (www.mn-net.com) following the manufacturers protocol. All 166 genomic DNA samples were genotyped using the Ovine 50 K SNP BeadChip assay. The assay includes 54,240 SNPs composed of 52,413 autosomal, 1449 X-chromosome and 378 mitochondrial SNPs, respectively.

**Table 1:** Description of the populations that were sampled for this study.

**Origin**	**Population**	**Zone**	**Latitude (*N*)**	**Longitude (E)**	**Altitude (m)**	***N***	**Tail type**	**Agro-ecology**
Ethiopia	Kefis	Zone 3	9°30′	40°10′	890	14	Fat-Rump	Arid lowland
	Adane	South Wollo	11°14′	39°50′	2,450	12	Fat-Rump	Cool highland
	Arabo	South Wollo	11°31′	36°54′	1,500	10	Fat-Rump	Cool highland
	Gafera-Washera	Agew Awi	11°31′	36°54′	2,500	15	Short, fat-tail	Wet, warmer mid-highland
	Molale-Menz	North Shewa	10°70′	39°39′	3,068	15	Short, fat-tail	Sub-alpine
	Bonga	Keffa	7°16′	36°15′	1,788	15	Long, fat-tail	Humid mid-highland
	Gesses	Metekel	10°50′	36°14′	1,300	10	Long, fat-tail	Moist lowlands
	Kido	Metekel	10°71′	36°19′	1,300	10	Long, fat-tail	Moist lowland
	Doyogena	Kembata Tembara	7°21′	37°47′	2,324	15	Long, fat-tail	Cool, wet highland
	ShubiGemo	East Shewa	8°80′	38°51	1,600	15	Long, fat-tail	Cool, wet highland
	Loya	Sidama	6°29′	38°24′	1,900	15	Long, fat-tail	Cool, wet highland
Sudan	Hammari	North Kurdufan	13°09′	29°22′	620	11	Long, thin-tail	Arid lowland
	Kabashi	North Kurdufan	13°09′	29°22′	620	9	Long, thin-tail	Arid lowland
Total						166		

Ovine 50 K SNP BeadChip genotypes of Caribbean, European, Middle East and Chinese, as well as western, northern and southern African sheep, respectively were obtained from the Sheep HapMap database (http://www.sheephapmap.org/hapmap.php, Supplementary Table 1) and included in the study. The aim was to provide a global context of the genetic origins, trajectories of introduction, and dispersal of sheep into Ethiopia.

### Quality Control and Genetic Diversity Analyses

The Sheep HapMap dataset were merged with the ones generated from Ethiopian and Sudanese sheep using PLINK v1.9 (Purcell et al., 2007). The data files for final analysis were generated after pruning the merged dataset of SNPs not mapping on any autosomes, with a minor allele frequency (MAF) ≤ 0.01 and animals and markers with ≥10 and 5% missing genotypes, respectively. This generated a dataset with 45,102 SNPs which were further pruned, using PLINK v1.9, to be in approximate linkage equilibrium to avoid the possible influence of clusters of SNPs on population genetic relationship and structure analysis (Yuan et al., 2017). Following the latter pruning, 34,088 SNPs were retained for population relatedness and structure analysis.

To minimize the possible loss of informative SNPs for selection signature analysis, the data for Ethiopian and Sudanese sheep was extracted from the dataset of 45,102 autosomal SNPs, that was obtained prior to LD pruning.

The proportion of polymorphic SNPs (*Pn*), expected (*He*), and observed (*Ho*) heterozygosity and inbreeding coefficient (*F*) were estimated for each population and across all populations using PLINK v1.9, to evaluate the levels of genetic diversity present in Ethiopian and Sudanese sheep, respectively.

### Population Genetic Analyses

Principal component analysis (PCA) were performed using PLINK v1.9 to investigate the genetic structure and relationships among the studied breeds based on genetic correlations between individuals. A graphical display of the first two principal components (PC1 and PC2) was generated using GENESIS (Buchmann and Hazelhurst, 2014). Admixture analysis implemented in ADMIXTURE v1.3 (Alexander et al., 2009) was used to investigate underlying genetic structure and estimate the proportion of shared genome ancestry between the study populations. A 5-fold cross-validation procedure following Lawal et al. (2018), was used to determine the optimal number of ancestral genomes (*K*) and proportions of genome ancestry that was shared among the study populations.

To further evaluate historical relationships and interactions (gene flow) within and between Ethiopian and Sudanese populations, we used the maximum likelihood tree-based approach implemented in TreeMix (Pickrell and Pritchard, 2012) and included the Soay sheep as an out-group. The number of migration events (m) varied between 1 (migration between two populations and 15 (migration between all the populations). The value of “m” with the highest reproducibility and consistency, among the 15 tested, and which also had the highest log-likelihood value following six replication runs of the analysis, was chosen as the most optimal.

The *f3* and *f4* tests implemented in TreeMix were also performed. The *f3*-statistics (A, B, C) were to determine if A was derived from the admixture of populations B and C; a significantly negative value of the *f3*-statistics would suggest population A is admixed. The *f4*-statistics (A, B,) (C, D) were to test the validity of hierarchical clustering pattern in four-population trees. Significant deviations of the *f4*-statistics from zero for the three possible topologies of the four-population trees would provide evidence of gene flow between the populations tested. A significantly positive *Z*-score indicates gene flow between populations that are related to either A and C or B and D while a significantly negative *Z*-score indicates gene flow between populations that are related to A and D or B and C. Standard errors were estimated using blocks of 500 SNPs.

### Analysis of Signatures of Selection

For this analysis, we separated 12 of the 13 Ethiopian and Sudanese populations into four genetic groups based on the population clusters revealed by PCA. The four population groups included, western (Bonga, Kido, Gesses) and southern (Loya, ShubiGemo, Doyogena) long fat-tail, and fat-rump (Kefis, Adane, Arabo) sheep from Ethiopia and thin-tail sheep (Hammari, Kabashi) from Sudan. One short fat-tail sheep (Molale) was included with the fat-rump sheep and the other (Gafera), which appeared to be genetically distinct, was dropped from further analysis. Equal numbers of samples were chosen at random to represent each genetic group. Three comparisons which contrasted the fat-rump (E1), western (E2) and southern (E3) long fat-tail sheep with the thin-tail sheep (S) from Sudan were performed. The selection signature analysis involved three approaches, *F*_*ST*_, *hapFLK* and *Rsb*.

A sliding window approach was used to perform the *F*_*ST*_ analysis using the HIERFSTAT package (Goudet, 2005) of R (R Core Team, 2012). The window size of 200 Kb, was allowed to slide along the genome by a distance of 60 Kb. The window size and slide distance were determined based on linkage disequilibrium (LD) decay analysis (Supplementary Figure 1). The pairwise *F*_*ST*_ values (Weir and Cockerham, 1984) for each SNP in each window and between the genetic groups being tested were estimated as follows:

$$\begin{array}{l} F_{ST}=1-\;\frac{p1q1+p2q2}{2prqr} \end{array}$$

Where *p1, p2* and *q1, q2* are the frequencies of alleles A and a in the first and second group of the test populations, respectively, and *pr* and *qr* are the frequencies of alleles A and a, respectively, across the tested groups (Zhi et al., 2018). The *F*_*ST*_ values were standardized into *Z*-scores as follows:

$$\begin{array}{l} ZF_{ST}=\frac{F_{ST}-\mu F_{ST}}{\sigma F_{ST}} \end{array}$$

Where μ*F*_*ST*_ is the overall average value of *F*_*ST*_ and σ*F*_*ST*_ is the standard deviation derived from all the windows tested for a given comparison. Supplementary Figure 2 shows the distribution of the *ZF*_*ST*_ values. We set the value of *ZF*_*ST*_ ≥ 4 as the threshold to identify candidate genomic regions under selection.

The *hapFLK* approach was implemented with *hapFLK* package v1.2 (Fariello et al., 2013) to detect selection signatures based on differences in haplotype frequencies between groups of populations. Reynolds genetic distances were converted into kinship matrix using an R script supplied with the package. The *hapFLK* values and kinship matrix were calculated assuming 15 clusters in the fastPHASE model (-K 15). The *hapFLK* statistic was then computed as the average value across 40 expectation maximization (EM) runs to fit the LD model. The *P*-values were obtained by running a python script “Scaling_chi^2^_hapFLK.py” available at (https://forge-dga.jouy.inra.fr/documents/588) which fits a chi-squared distribution to the empirical distribution. As with the *F*_*ST*_ calculations, the *hapFLK* statistics were also standardized using the formula:

$$\begin{array}{l} hapFLKadj=\;\frac{hapFLK\text{\_}mean\left(hapFLK\right)}{Sd\left(hapFLK\right)} \end{array}$$

The calculation of the raw *P*-values was based on the null distribution of empirical values (Fariello et al., 2013; Kijas, 2014). The *P*-values were plotted in a histogram to assess their distribution pattern and the cut-off value to determine significance was set at –Log10 (*P*-value) ≥ 3 (Supplementary Figure 2).

Using haplotype information, we performed the *Rsb* analysis implemented in *rehh* package (Gautier and Vitalis, 2012) of R. Haplotypes were estimated with SHAPEIT (Delaneau et al., 2014). To identify loci under selection, the *Rsb* values were log-transformed into *P*_*Rsb*_ (*P*_*Rsb*_ = –Log10 [1–2(Φ (*Rsb*)−0, 5)]), where Φ(x) represents the Gaussian cumulative distribution function (Gautier and Vitalis, 2012). Assuming that the *Rsb* values are normally distributed (under neutrality), the *P*_*Rsb*_ can be interpreted as –Log10 (*P*-value), where *P* is the two-sided *P*-value associated with the neutral hypothesis. For each comparison, SNPs that exhibited *P*_*Rsb*_ ≥ 3 (*P*-value = 0.001) were taken to be under selection (de Simoni Gouveia et al., 2017). The *hapFLK* and *Rsb* analysis were also performed using window sizes of 200 Kb sliding along the genome by a distance of 60 Kb.

### Gene Annotation

Candidate regions that overlapped between *F*_*ST*_, *hapFLK*, and *Rsb* were identified and compared using the intersectBed function of Bed Tools software (Quinlan and Hall, 2010). Considering an average marker distance of between 60 and 200 Kb (Moioli et al., 2015) and the observed LD decay pattern (Supplementary Figure 1), candidate regions under selection were identified by exploring the SNPs found up- and down-stream, and within, the most significant windows. The Oar v3.1 Ovine reference genome assembly (Jiang et al., 2014) was used to annotate the candidate regions. Functional enrichment analysis was performed using the functional annotation tool in *DAVID* (Huang et al., 2008) using *Ovis aries* as the background species. Gene functions were determined using the NCBI (http://www.ncbi.nlm.nih.gov/gene/) and OMIM databases (http://www.ncbi.nlm.nih.gov/omim/) and a review of literature.

## Results

### Genetic Diversity and Population Structure

The average values of *Pn, He, Ho*, and *F*, as indicators of within-breed genetic diversity, are shown in Table 2 and Supplementary Figure 3. The lowest values of *Pn, He*, and *Ho* were observed in Bonga while the highest values were observed in Molale-Menz, Hammari and Kabashi, and Arabo, respectively.

**Table 2:** Measures of genetic diversity for each of the 13 populations analyzed.

**Breed**	***N***	***P_***n***_*(%)**	***H_***e***_***	***H_***o***_***	***F***
Kefis	14	89.95	0.316	0.328	0.035
Adane	12	88.85	0.315	0.319	0.071
Arabo	10	88.69	0.317	0.334	0.050
Molale-Menz	15	90.29	0.316	0.319	0.055
Gafera-Washera	15	87.54	0.303	0.318	0.017
Bonga	9	79.59	0.277	0.293	0.038
Kido	10	82.18	0.290	0.310	0.038
Gesses	10	83.09	0.294	0.317	0.027
Doyogena	15	87.17	0.302	0.308	0.044
Loya	15	83.58	0.286	0.294	0.039
ShubiGemo	15	88.40	0.304	0.313	0.037
Hammari	11	89.93	0.319	0.332	0.038
Kabashi	9	88.64	0.319	0.328	0.025

**Figure 2:**
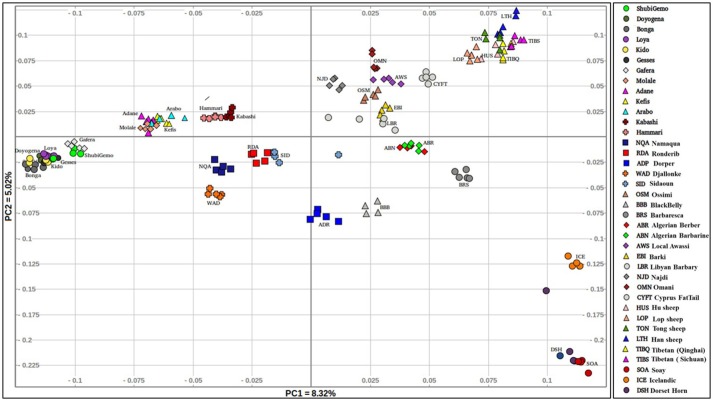
Genetic variation among the Ethiopian sheep populations in a global geographic context.

The PCA plot incorporating the global populations and which was constructed using a sample size of five animals that were selected at random per population, is shown in Figure 2. We used the uniform sample size of five animals since differences in sample sizes may influence clustering patterns on the PCA. The choice to use five samples per population was based on the smallest sample size of five individuals genotyped for Sidaoun and Berber breeds. In spite the sample size rebalancing, the population cluster patterns did not differ from that observed when the PCA was performed using unequal sample sizes (Supplementary Figures 4, 5). Generally, PC1 separates Ethiopian and South African fat-tail sheep, Sudanese thin-tail sheep, West African Djallonke and Algerian Sidaoun from the other sheep populations. Sheep from the Middle East and North Africa occur at the center of the PCA plot and, together with the Cyprus fat-tail and Chinese sheep (which cluster close together) are separated by PC2 from African Dorper, Barbados Blackbelly and European sheep. The two populations of Ethiopian short fat-tail sheep diverge from each other; Gafera-Washera clusters near Ethiopian long fat-tail sheep while Molale-Menz clusters together with the Ethiopian fat-rump sheep. The West African Djallonke clusters close to the two South African breeds (Ronderib and Namaqua). Sidaoun and Berber (both from Algeria) cluster separate, while the Cyprus fat-tail clusters close to the Chinese sheep (Figure 2).

**Figure 3:**
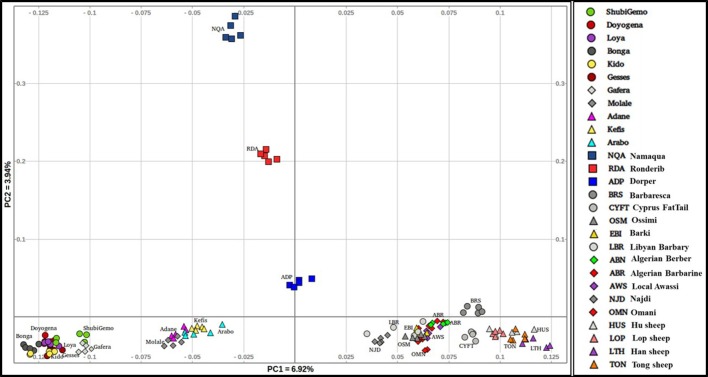
Distribution of genetic variation among the worldwide fat-tail sheep.

To obtain a clearer picture of the variation within the fat-tail sheep, we performed the PCA excluding the thin-tail sheep (Figure 3). PC1 separates the Ethiopian fat-tails from their Middle East, North Africa, Mediterranean and Chinese counterparts. PC2 differentiates the South African breeds from the Ethiopian ones. Like the global PCA, one Ethiopian short fat-tail sheep (Gafera-Washera) clusters with the Ethiopian long-fat tail sheep and the other (Molale-Menz) forms a cluster with the Ethiopian fat-rump sheep. Middle East sheep cluster together with the North African ones while the Mediterranean sheep unexpectedly cluster with the Chinese sheep despite the large geographic distance separating them.

**Figure 4:**
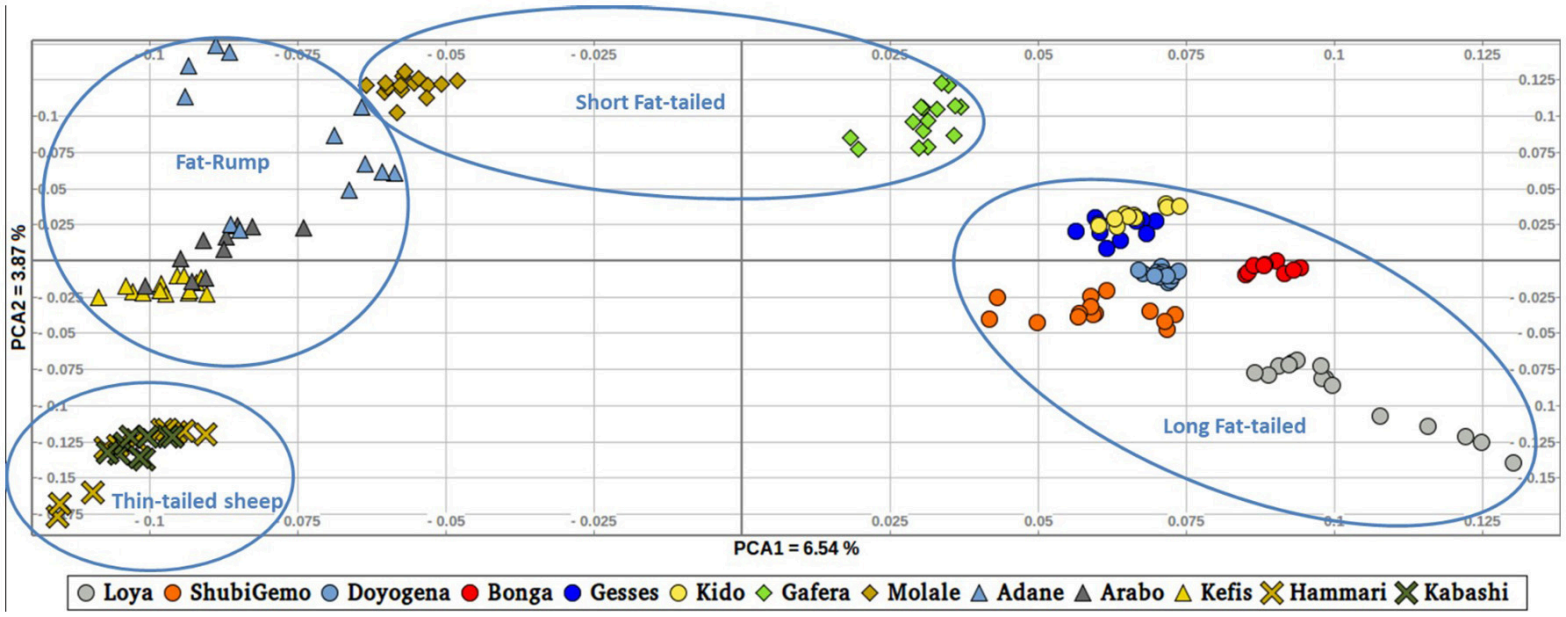
Distribution of genetic variation among the East African sheep populations (PC1 and PC2).

To further illustrate the distribution of genetic variation among East African populations, we performed the PCA with only the Ethiopian and Sudanese thin-tail sheep (Figure 4). PC1 separates Ethiopian fat-rump, Molale-Menz (Ethiopian short-fat tail) and thin-tail sheep from the Ethiopian long fat-tail and Gafera-Washera (Ethiopian short-fat tail) sheep. Generally, PC1 separates the fat-rump sheep from the fat-tail ones derived from western and southern Ethiopia. PC2 reveals further separation of the Ethiopian sheep: (i) Molale-Menz, Adane and some Arabo individuals from Kefis, and the remaining Arabo individuals, and (ii) Gafera-Washera, Kido and Gesses from Doyogena, ShubiGemo, Bonga and Loya.

**Figure 5:**
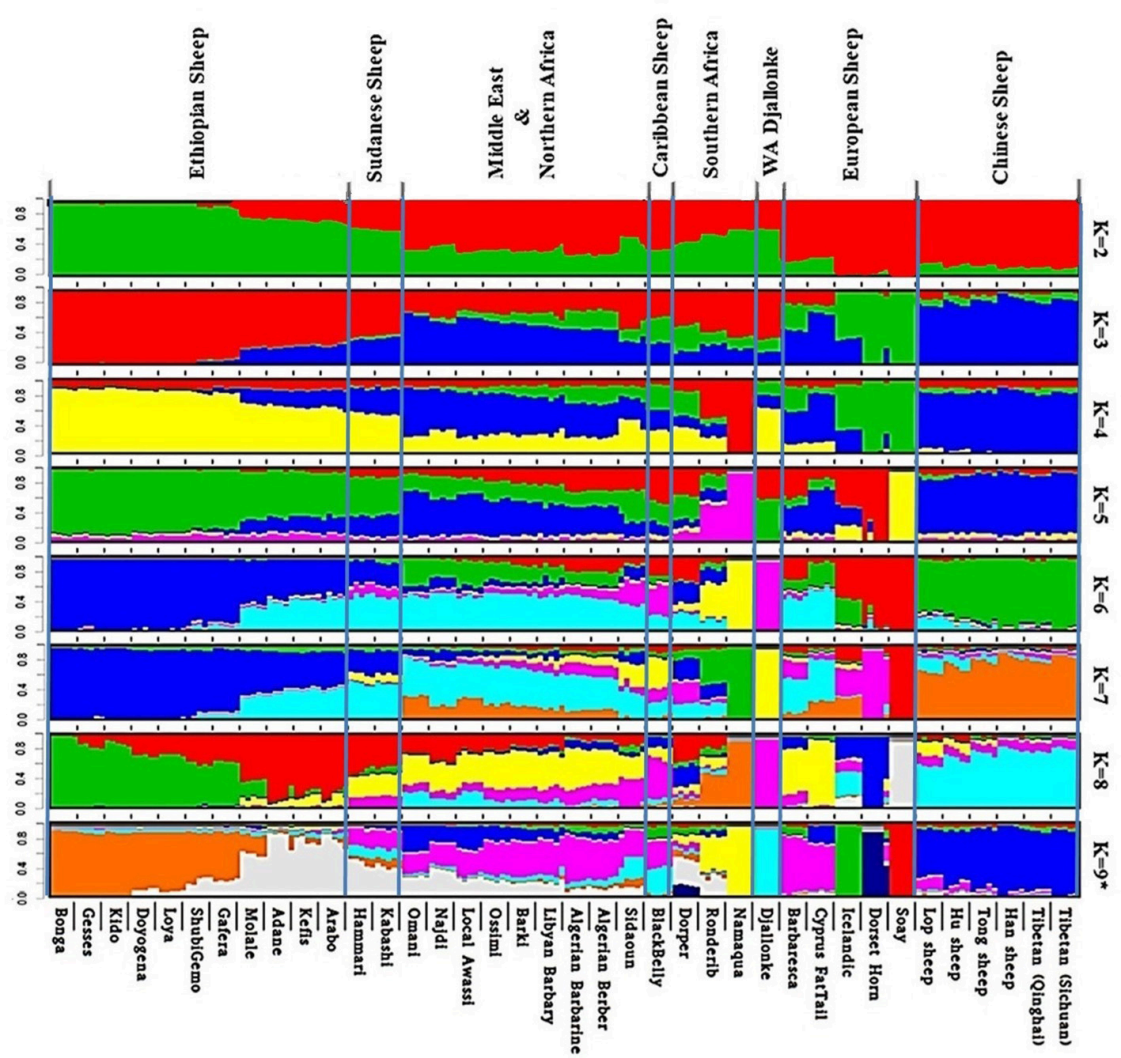
Admixture analysis of the studied populations in a global context (*K* = 9 had the lowest cross-validation error).

Admixture analysis on the global dataset, separates the study populations following their geographic origins (Figure 5). The cross-validation error registered the lowest value at *K* = 9 suggesting this to be the most optimal number of clusters explaining the variation in this dataset (Supplementary Figure 6a). Chinese sheep separate from the other populations at *K* ≥ 3. Among African breeds, the South African ones (Namaqua, Dorper, Ronderib) and the West African Djallonke show a distinct but common genetic ancestry with the Ethiopian and Sudanese sheep for 3 ≤ *K* ≤ 6.

**Figure 6:**
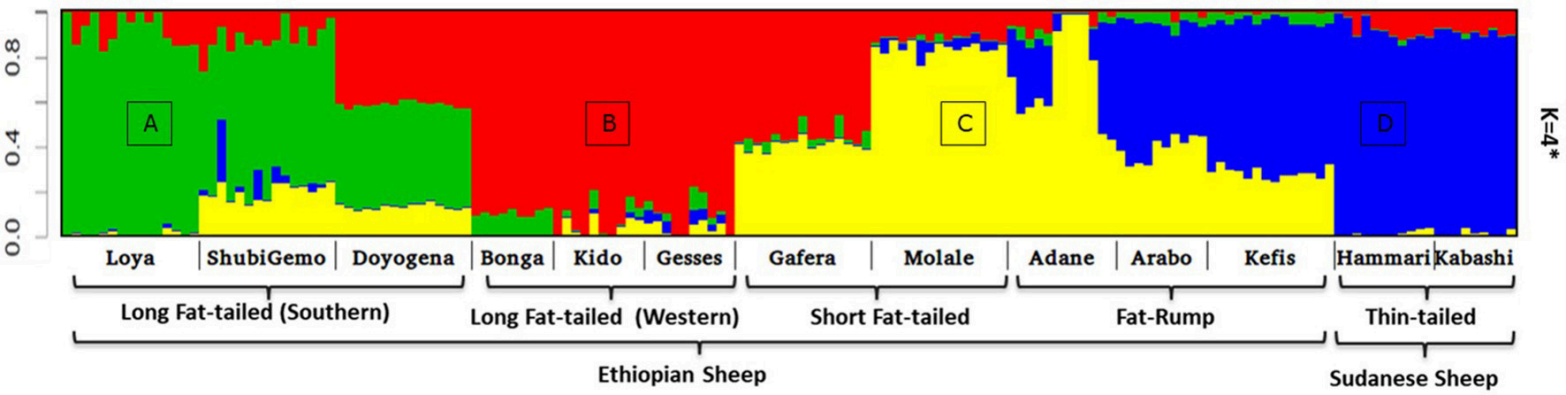
Admixture analysis involving Ethiopian indigenous sheep populations (*K* = 4 had the lowest cross-validation error). For brevity the four genetic clusters are designated **(A)**–**(D)**, respectively.

Two to six hypothetical ancestral clusters (*K*) were tested with Admixture on the East African dataset. The lowest cross-validation error suggests *K* = 4 (Supplementary Figure 6b) as the optimal number of ancestral clusters present in Ethiopian and Sudanese thin-tail sheep. The proportion of each ancestral cluster (referred to as A, B, C, and D) in each population at *K* = 4 is shown in Figure 6 and Supplementary Table 2. They occur with the highest proportion (>90%) in Loya (cluster A), Bonga, Kido and Gesses (cluster B), Molale-Menz and a few individuals of Adane (cluster C) and in thin-tail sheep (cluster D). Clusters A, B, and C are observed in ShubiGemo and Doyogena; B and C in Gafera-Washera and Molale-Menz; B, C, and D in some individuals of Adane while Arabo and Kefis had C and D clusters. The analysis also shows that Gafera-Washera, Adane, Molale-Menz, Arabo, and Kefis share cluster C, while Hammari and Kabashi share the D cluster with Arabo and Kefis. ShubiGemo, Loya and Doyogena, all long fat-tail sheep from southern Ethiopia, share cluster A.

**Figure 7:**
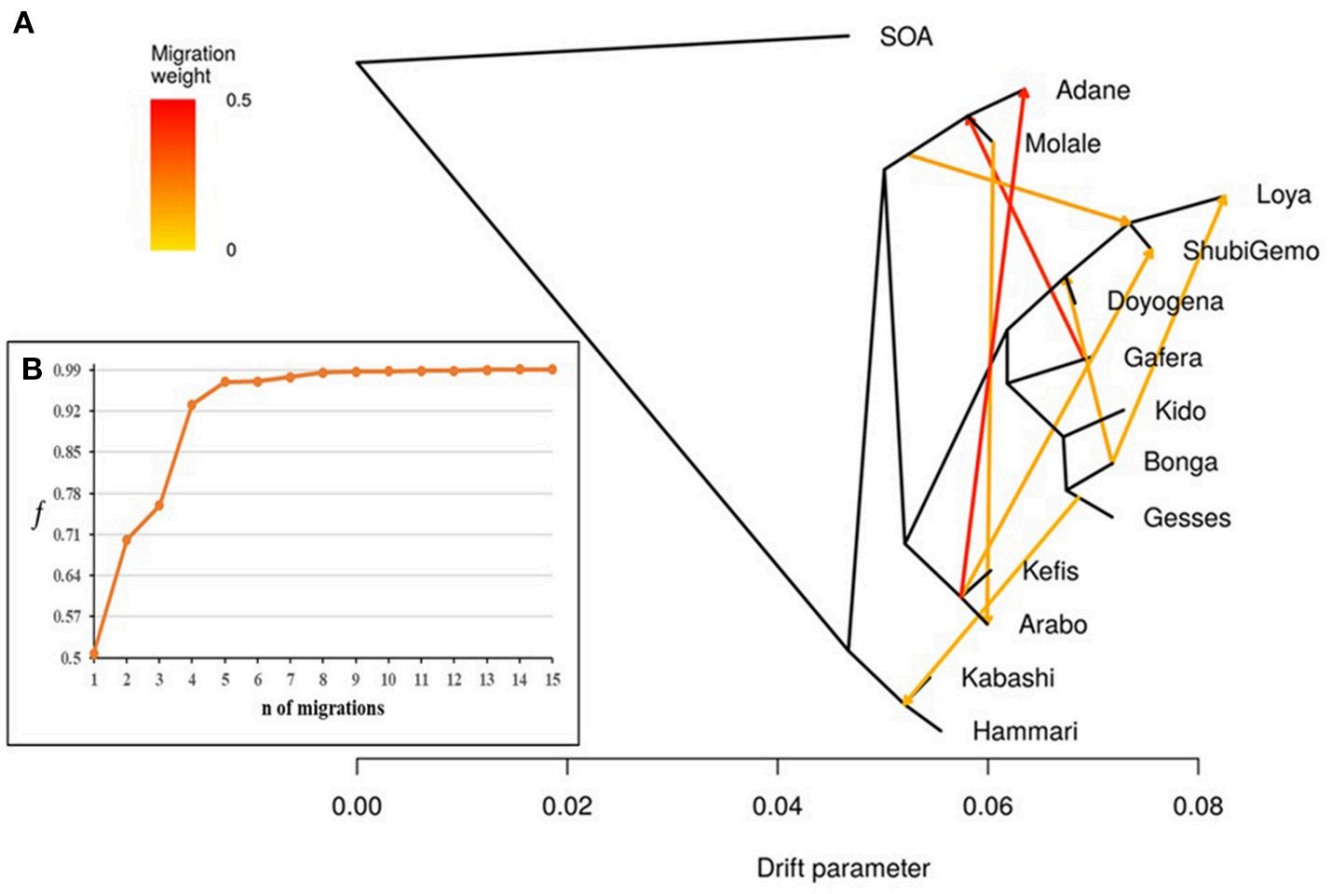
Tree-mix plot. **(A)** Phylogenetic network inferred by Tree-mix of the relationships between Ethiopian and Sudanese sheep populations. The first eight migration edges between populations are shown with arrows pointing in the direction toward the recipient group and colored according to the ancestry percentage received from the donor. **(B)** Shows the *f* index representing the fraction of the variance in the sample covariance matrix ($\hat{\text{W}}$) accounted for by the model covariance matrix (W), as a function of the number of modeled migration events.

TreeMix revealed possibilities of gene-flow between East African sheep. The *f* index representing the fraction of the variance in the sample covariance matrix (Ŵ) accounted for by the model covariance matrix (W) was used to identify the information contribution of each migration vector added to the tree. Up to 15 possible migration vertices were computed. The first eight migration edges (gene flow) accounted for more than half of the total model significance explained by the *f* statistic, with the first migration edge having a *f* value of 0.51. We therefore chose *m* = 8 as the best predictive value for the migration model. Vectors from 9 to 15 resulted in only small incremental changes in the *f* value (Figures 7A,B). The eight migration events were Loya and ShubiGemo (both long fat-tail); Arabo and Adane (both fat-rump); Gafera-Washera, Molale-Menz (both short fat-tail) and Adane (fat-rump); Molale-Menz (short fat-tail) and Adane (fat-rump) with ShubiGemo (long fat-tail); Bonga with ShubiGemo, Doyogena and Loya (all long fat-tail sheep); Molale-Menz (short fat-tail) and Arabo (fat-rump); ShubiGemo (long fat-tail) with Arabo (fat-rump) and Kefis (fat-rump); Gesses (long fat-tail) with Kabashi and Hammari (thin-tail).

The *f4*-statistics, also highlighted possibilities of gene flow among various breeds. The highest *Z* values (>|50|) were observed between Hammari and Kabashi (thin-tails) and Arabo and Kefis (fat-rump) (Supplementary Table 3). The *f3-*statistics however, did not reveal any likelihood of gene-flow between the breeds analyzed (Supplementary Table 4). This could be due to a complex pattern of gene-flow between the study populations, which may not be accounted for by a three-way model.

### Signatures of Selection

The Admixture, TreeMix and PCA (Figures 6, 7; Supplementary Figure 4) revealed three genetic groups in Ethiopian sheep *viz* fat-rump (E1), and long fat-tail from western (E2) and southern (E3) Ethiopia, respectively. The two short fat-tail sheep (Molale-Menz and Gafera-Washera) analyzed here were separated from each other (Figure 4) with Molale-Menz showing close genetic affinity to fat-rump sheep and Gafera-Washera appeared genetically distinct. The three groups are distinct from thin-tail (S) sheep (Figure 4). For selection signature analysis, we included Molale-Menz with the fat-rump sheep but excluded Gafera-Washera from the analysis due to its low sample size. We selected, at random, 20 samples to represent each of the four genetic groups and performed the selection signature analysis. We contrasted the three groups of Ethiopian sheep (E1, E2, and E3) with the thin-tail sheep (S). The top windows (Supplementary Table 5), which passed the significance threshold, for each method (*hapFLK* ≥ 3, *ZF*_*ST*_ ≥ 4, *Rsb* ≥ 3) were used to define candidate regions under selection.

**Figure 8:**
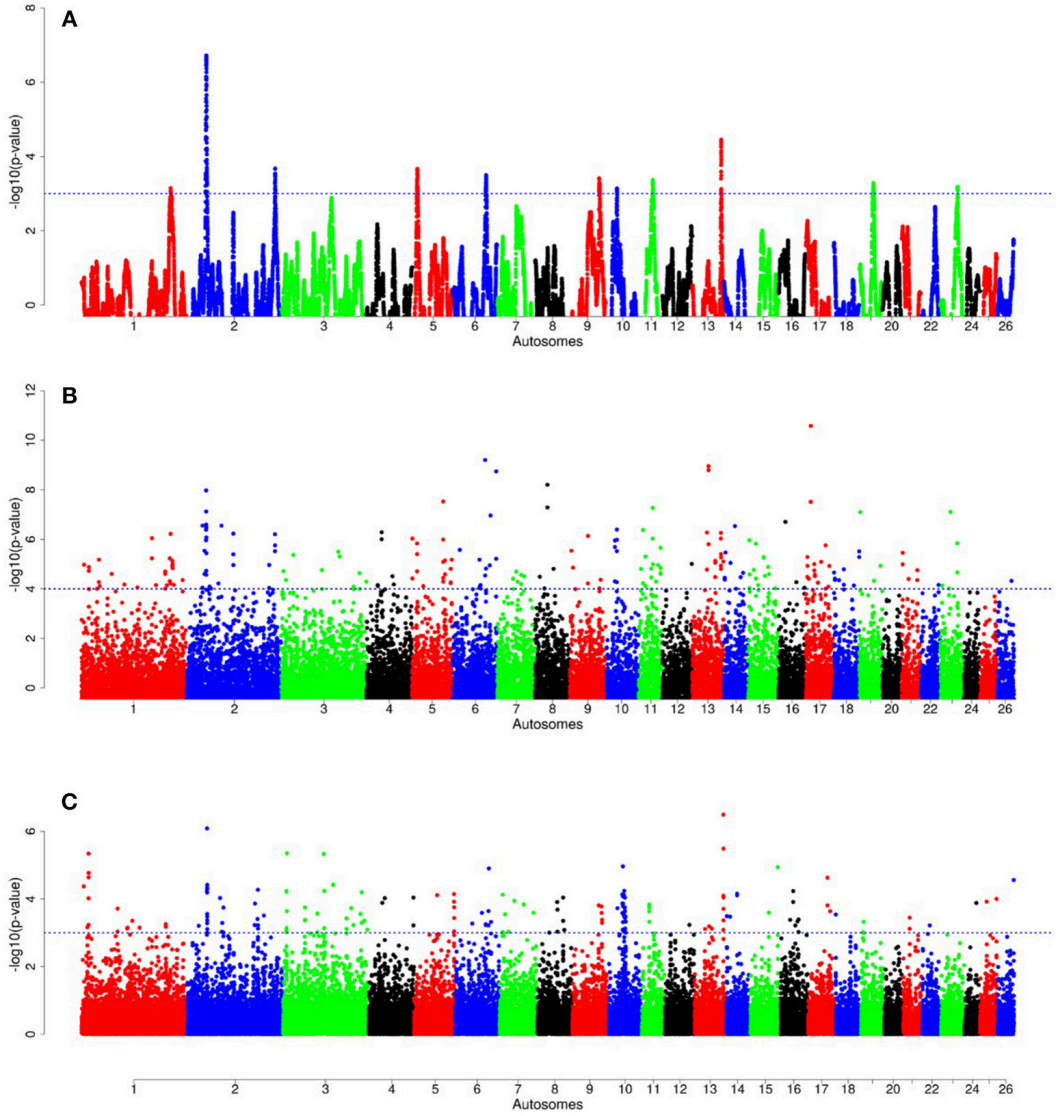
Manhattan plots of genome-wide autosomal *hapFLK* **(A)**, *ZF*_*ST*_ **(B)** and *RsB* **(C)** analyses of Ethiopian fat-rump (E1) *vs*. thin-tail (S) sheep.

**Figure 9:**
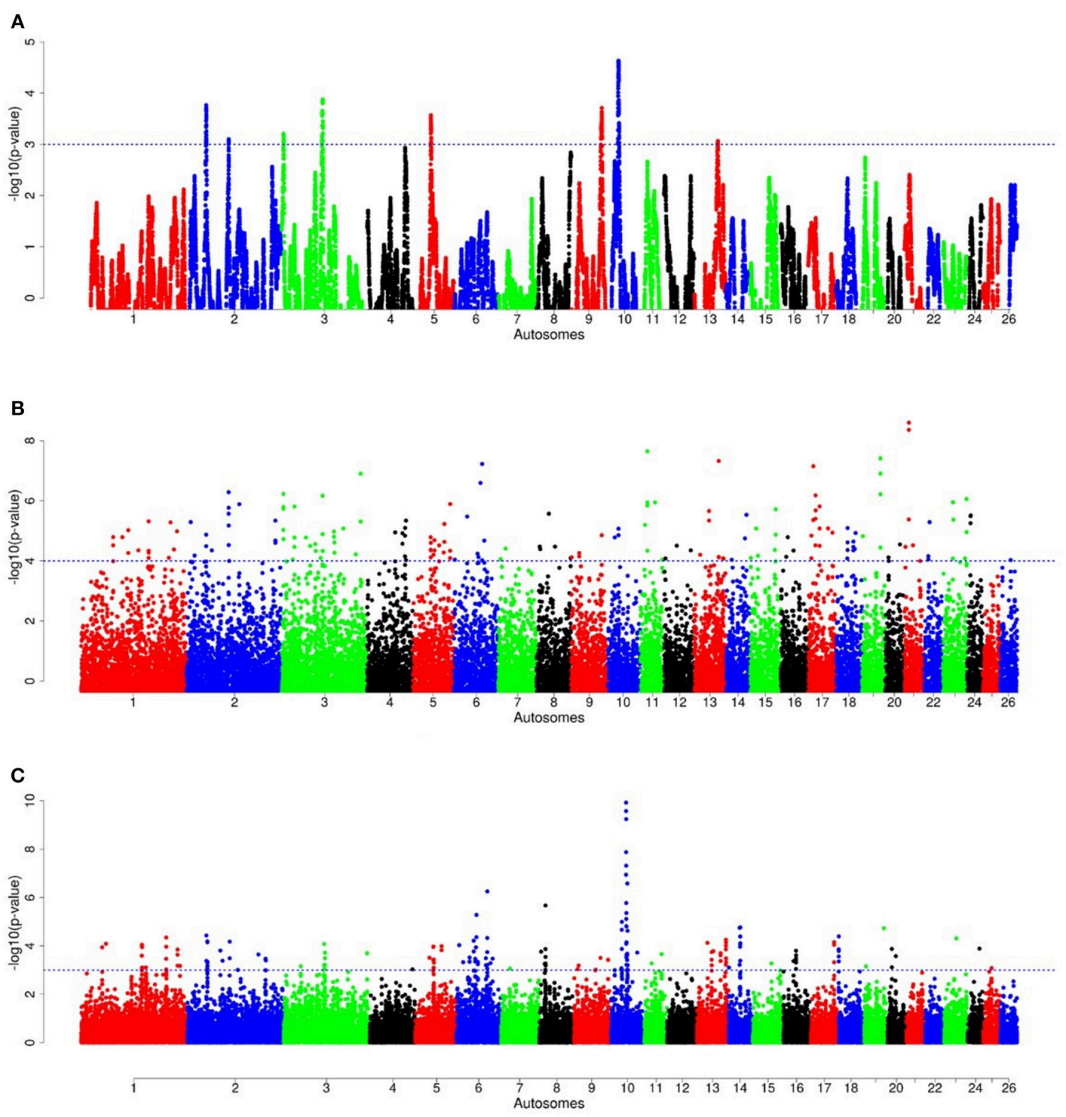
Manhattan plots of genome-wide autosomal *hapFLK* **(A)**, *ZF*_*ST*_ **(B)** and *RsB* **(C)** analyses of western Ethiopian long fat-tail sheep (E2) vs. thin-tail (S) sheep.

**Figure 10:**
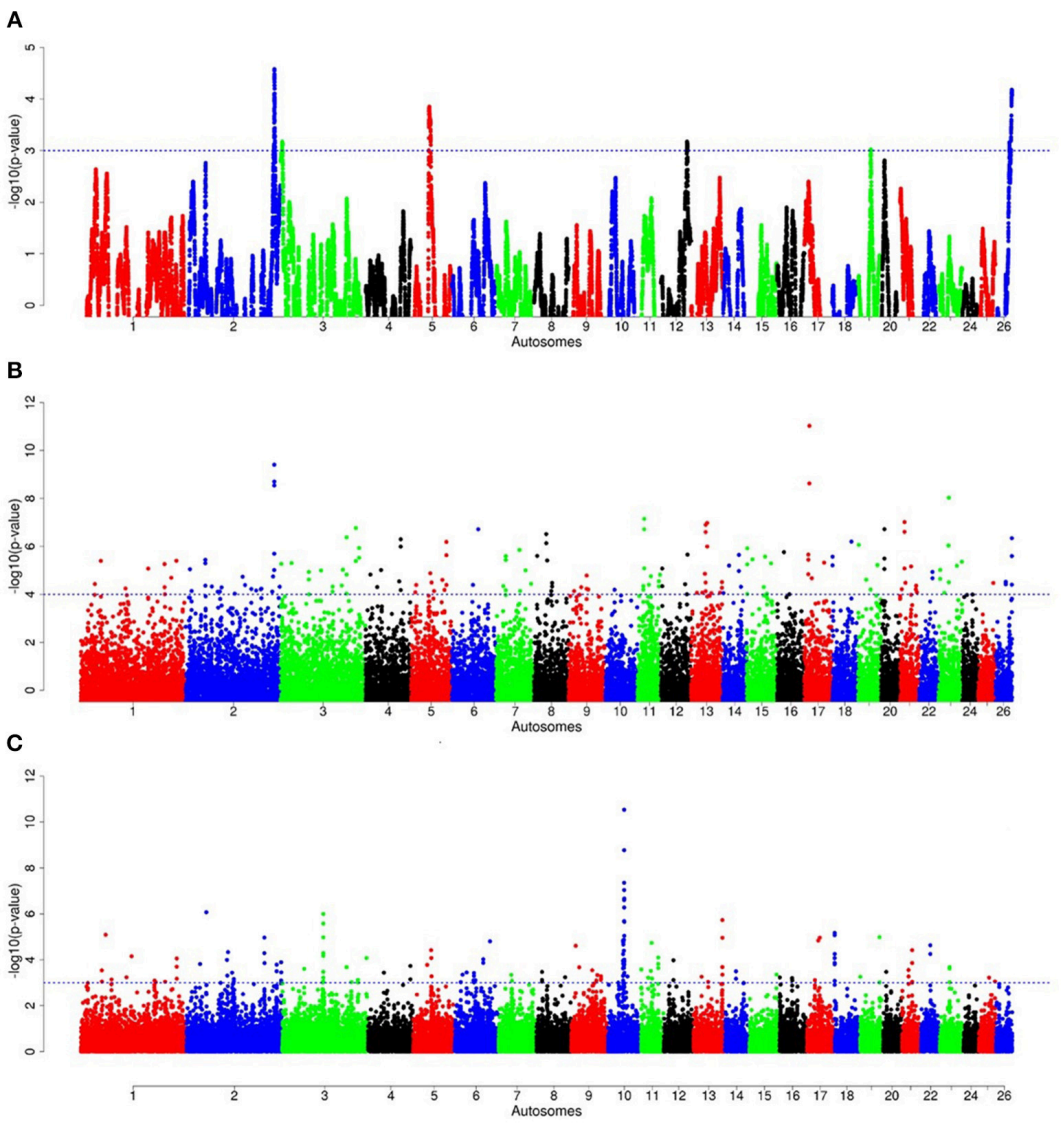
Manhattan plots of genome-wide autosomal *hapFLK* **(A)**, *ZF*_*ST*_ **(B)** and *RsB* **(C)** analyses of southern Ethiopian long fat-tail (E3) vs. thin-tail (S) sheep.

For E1^*^S comparison, the fat-rump sheep were differentiated from the thin-tail in 23 candidate regions that overlapped between at least two selection signature methods and which spanned 86 genes (Figure 8, Table 3). Similarly, a total of 65 genes were present across 18 candidate regions that overlapped between at least two approaches in the E2^*^S (western Ethiopia long fat-tail verses thin-tail) comparison (Figure 9, Table 4). Furthermore, 10 genes that seemed to be highly selected were identified by *Rsb* in three candidate regions on Oar8, Oar14, and Oar18, respectively (Figure 9, Table 4). Twelve overlapping candidate regions spanning 36 genes, were observed in the southern Ethiopian fat-tail verses thin-tail sheep (E3^*^S) (Figure 10, Table 5). There were also 16 genes found across 1 (Oar26, 3 genes), 1 (Oar3, 1 gene), and 12 (Oar2, 1 gene; Oar3, 9 genes; Oar10, 2 genes) candidate regions that were identified by *hapFLK, ZF*_*ST*_, and *Rsb*, respectively (Figure 10, Table 5).

**Table 3:** Candidate regions and genes identified to be under selection by a combination of at least two methods in the Ethiopian fat-rump vs. Sudanese thin-tail sheep.

**Chr**	**Overlapping region**	**Gene location**	**Method**	**Candidate gene**	**Annotation**
1	6600001-6800000	6767995-6787884	Rsb*ZF_*ST*_	SPP2	secreted phosphoprotein 2
	19200001-19460000	19196066-19216520		KIF2C	kinesin family member 2C
		19227521-19230058		RPS8	ribosomal protein S8
		19233256-19236856		BEST4	bestrophin 4
		19251263-19255855		PLK3	polo like kinase 3
		19256190-19256846		TCTEX1D4	Tctex1 domain containing 4
		19258856-19263210		BTBD19	BTB domain containing 19
		19270475-19284541		PTCH2	patched 2
		19291159-19423029		EIF2B3	eukaryotic translation initiation factor 2B subunit gamma
		19425022-19433738		HECTD3	HECT domain E3 ubiquitin protein ligase 3
		19436350-19439595		UROD	uroporphyrinogen decarboxylase
		19442382-19608681		ZSWIM5	zinc finger SWIM-type containing 5
2	51660001-52220000	51686233-51755300	hapFLK*ZF_*ST*_	MELK	maternal embryonic leucine zipper kinase
		51891433-51948694		RNF38	ring finger protein 38
		51989342-52042116		GNE	glucosamine (UDP-N-acetyl)-2-epimerase/N-acetylmannosamine kinase
		52048202-52065307		CLTA	clathrin light chain A
		52087650-52089416		CCIN	Calicin
		52128947-52210749		RECK	reversion inducing cysteine rich protein with kazal motifs
	52020001-53180000	52411021-52417200	hapFLK*Rsb*ZF_*ST*_	FAM221B	family with sequence similarity 221 member B
		52421111-52423389		HINT2	histidine triad nucleotide binding protein 2
		52423298-52426475		SPAG8	sperm associated antigen 8
		52423842-52445175		NPR2	natriuretic peptide receptor 2
		52480200-52481163		MSMP	microseminoprotein, prostate associated
		52480334-52485038		RGP1	RGP1 homolog, RAB6A GEF complex partner 1
		52485320-52495944		GBA2	glucosylceramidase beta 2
		52496387-52500153		CREB3	cAMP responsive element binding protein 3
		52506528-52531560		TLN1	talin 1
		52537459-52544952		TPM2	tropomyosin 2
		52546134-52551851		CA9	carbonic anhydrase 9
		52560703-52563910		ARHGEF39	Rho guanine nucleotide exchange factor 39
		52564548-52567161		CCDC107	coiled-coil domain containing 107
		52572730-52573775		SIT1	signaling threshold regulating transmembrane adaptor 1
		52594675-52607206		CD72	CD72 molecule
		52603605-52607846		TESK1	testis-specific kinase 1
		52616756-52618641		FAM166B	family with sequence similarity 166 member B
		52619243-52632387		RUSC2	RUN and SH3 domain containing 2
		52817902-53036532	hapFLK*Rsb	UNC13B	unc-13 homolog B
		53056098-53059144		FAM214B	family with sequence similarity 214 member B
		53061224-53067598		STOML2	stomatin like 2
		53070391-53079125		PIGO	phosphatidylinositol glycan anchor biosynthesis class O
		53079030-53084363		FANCG	Fanconi anemia complementation group G
		53089776-53099744		VCP	valosin containing protein
		53138867-53146827		DNAJB5	DnaJ heat shock protein family (Hsp40) member B5
		53159612-53165463		PHF24	PHD finger protein 24
	232620001-232940000	232749221-233048136	hapFLK*ZF_*ST*_	DIS3L2	DIS3 like 3'-5' exoribonuclease 2
3	107100001-107240000	107108271-107174474	Rsb*ZF_*ST*_	TSPAN8	tetraspanin 8
	205800001-206000000	205801838-205853818		A2ML1	alpha-2-macroglobulin like 1
		205889722-205909753		RIMKLB	ribosomal modification protein rimK like family member B
		205954117-205968927		MFAP5	microfibril associated protein 5
		205985865-205999107		AICDA	activation induced cytidine deaminase
5	13620001-13940000	13733596-13879145	hapFLK*ZF_*ST*_	INSR	insulin receptor
6	70200001-70520000	70189729-70234612	Rsb*ZF_*ST*_	KIT	KIT proto-oncogene receptor tyrosine kinase
	87180001-87560000	87097877-87386270	hapFLK*Rsb	ADAMTS3	ADAM metallopeptidase with thrombospondin type 1 motif 3
7	63420001-63620000	63450344-63456226	Rsb*ZF_*ST*_	BMP4	bone morphogenetic protein 4
9	76740001-77300000	76741376-76818820	hapFLK*Rsb	SPAG1	sperm associated antigen 1
		76826125-76827336		POLR2K	RNA polymerase II subunit K
		76838577-76849876		FBXO43	F-box protein 43
		76870092-77006581		RGS22	regulator of G protein signaling 22
		77057424-77839842		VPS13B	vacuolar protein sorting 13 homolog B
	78000001-78380000	78104905-78377671	hapFLK*ZF_ST_*Rsb	STK3	serine/threonine kinase 3
10	24240001-24500000	24289442-24435384	Rsb*ZF_*ST*_	TRPC4	transient receptor potential cation channel subfamily C member 4
		24474862-24508794		POSTN	periostin
	29400001-29780000	29454677-29502617	hapFLK*ZF_*ST*_	RXFP2	relaxin family peptide receptor 2
11	37140001-37400000	37140993-37148353		SNF8	SNF8, ESCRT-II complex subunit
		37146942-37164597		UBE2Z	ubiquitin conjugating enzyme E2 Z
		37173130-37175267		ATP5MC1	ATP synthase membrane subunit c locus 1
		37227823-37243185		CALCOCO2	calcium binding and coiled-coil domain 2
		37272426-37302473		TTLL6	tubulin tyrosine ligase like 6
		37337231-37338988		HOXB13	homeobox B13
	37920001-38120000	37924394-37928175		SNX11	sorting nexin 11
		37972076-37981743		NFE2L1	nuclear factor, erythroid 2 like 1
		37992980-38001708		COPZ2	coatomer protein complex subunit zeta 2
		38037788-38047808		CDK5RAP3	CDK5 regulatory subunit associated protein 3
		38063059-38063361		PRR15L	proline rich 15 like
		38069220-38075491		PNPO	pyridoxamine 5'-phosphate oxidase
		38082581-38118204		SP2	Sp2 transcription factor
13	38580001-38660000	38609366-38671551	Rsb*ZF_*ST*_	RIN2	Ras and Rab interactor 2
	75120001-75680000	75066765-75328455	hapFLK*ZF_ST_*Rsb	EYA2	EYA transcriptional coactivator and phosphatase 2
		75666854-75730764		NCOA3	nuclear receptor coactivator 3
		75726734-75771128	hapFLK*Rsb	SULF2	sulfatase 2
14	2220001-2360000	2251815-2262220	Rsb*ZF_*ST*_	GABARAPL2	GABA type A receptor associated protein like 2
		2276128-2300712		ADAT1	adenosine deaminase, tRNA specific 1
		2302972-2319972		KARS	lysyl-tRNA synthetase
	28860001-29000000	28747069-29125550		CDH8	cadherin 8
15	72540001-72620000	72556058-72606253	Rsb*ZF_*ST*_	ALX4	ALX homeobox 4
17	51780001-51800000	51771124-51788976	Rsb*ZF_*ST*_	RILPL2	Rab interacting lysosomal protein like 2
				

**Table 4:** Candidate regions and genes identified to be under selection by a combination of at least two methods in the Ethiopian western long fat-tail vs. Sudanese thin-tail sheep.

**Chr**	**Overlapping region**	**Gene location**	**Method**	**Candidate gene**	**Annotation**
2	51960001-52880000	51989342-52042116	hapFLK*Rsb	GNE	glucosamine (UDP-N-acetyl)-2-epimerase/N-acetylmannosamine kinase
		52048202-52065307		CLTA	clathrin light chain A
		52087650-52089416		CCIN	Calicin
		52128947-52210749		RECK	reversion inducing cysteine rich protein with kazal motifs
		52411021-52417200		FAM221B	family with sequence similarity 221 member B
		52421111-52423389		HINT2	histidine triad nucleotide binding protein 2
		52423298-52426475		SPAG8	sperm associated antigen 8
		52423842-52445175		NPR2	natriuretic peptide receptor 2
		52480200-52481163		MSMP	microseminoprotein, prostate associated
		52480334-52485038		RGP1	homolog, RAB6A GEF complex partner 1
	51720001-51980000	51686233-51755300	hapFLK*ZF_*ST*_	MELK	maternal embryonic leucine zipper kinase
		51891433-51948694		RNF38	ring finger protein 38
	110280001-110780000	110280423-110367262	hapFLK*ZF${}_{ST}^{*}$Rsb	CLCN3	chloride voltage-gated channel 3
		110404240-110525395		NEK1	NIMA related kinase 1
3	105360001-106220000	105840829-105932962	hapFLK*Rsb	ANAPC1	anaphase promoting complex subunit 1
		105945465-106063047		MERTK	MER proto-oncogene, tyrosine kinase
		106081347-106128188		TMEM87B	transmembrane protein 87B
		106141259-106197636		FBLN7	fibulin 7
	106860001-107240000	107108271-107174474	hapFLK*ZF${}_{ST}^{*}$Rsb	TSPAN8	tetraspanin 8
	4800001-5240000	5038319-5152424	hapFLK*ZF_*ST*_	RAPGEF1	Rap guanine nucleotide exchange factor 1
		5207016-5299854		UCK1	uridine-cytidine kinase 1
		5212765-5239836		POMT1	protein O-mannosyltransferase 1
	107580001-107840000	107556327-107605339	hapFLK*ZF_*ST*_	ZFC3H1	zinc finger C3H1-type containing
		107606256-107618590		THAP2	THAP domain containing 2
		107630616-107646736		TMEM19	transmembrane protein 19
		107781187-107834198		TBC1D15	TBC1 domain family member 15
5	46320001-46700000	46440670-46557263	hapFLK*ZF${}_{ST}^{*}$Rsb	KLHL3	kelch like family member 3
		46579802-46580796		HNRNPA0	heterogeneous nuclear ribonucleoprotein A0
	46740001-47120000	46741353-46780919		PKD2L2	polycystin 2 like 2, transient receptor potential cation channel
		46784304-46868016		FAM13B	family with sequence similarity 13 member B
		46910528-46915217		WNT8A	Wnt family member 8A
		46938902-46961998		NME5	NME/NM23 family member 5
		46972869-46993265		BRD8	bromodomain containing 8
		46994290-47001867		KIF20A	kinesin family member 20A
		47003373-47022642		CDC23	cell division cycle 23
		47062627-47080160		GFRA3	family receptor alpha 3
	47160001-47660000	47153096-47208649	hapFLK*Rsb	KDM3B	lysine demethylase 3B
		47209138-47212067		REEP2	receptor accessory protein 2
		47225278-47227722		EGR1	early growth response 1
		47292664-47309664		HSPA9	heat shock protein family A (Hsp70) member 9
		47473181-47642282		CTNNA1	catenin alpha 1
		47580182-47582084		LRRTM2	leucine rich repeat transmembrane neuronal 2
		47651357-47849675		SIL1	nucleotide exchange factor
	48060001-48140000	48060550-48066158		SPATA24	spermatogenesis associated 24
		48074043-48099657		DNAJC18	DnaJ heat shock protein family (Hsp40) member C18
		48118011-48122197		SMIM33	small integral membrane protein 33
		48123808-48127851		TMEM173	transmembrane protein 173
8	15780001-16700000	15790630-15823674	*Rsb*	SERINC1	serine incorporator 1
		15831623-15870470		HSF2	heat shock transcription factor 2
10	29700001-30320000	29893792-30043083	hapFLK*Rsb	B3GLCT	beta 3-glucosyltransferase
		30044800-30065505		HSPH1	heat shock protein family H (Hsp110) member 1
		30217152-30243100		TEX26	testis expressed 26
		30250695-30265933		MEDAG	mesenteric estrogen dependent adipogenesis
	29100001-29420000	28986741-29188660		FRY	FRY microtubule binding protein
	29280001-29540000	29454677-29502617	hapFLK*ZF_*ST*_	RXFP2	relaxin family peptide receptor 2
13	61320001-61700000	61459737-61515972	hapFLK*Rsb	DNMT3B	DNA methyltransferase 3 beta
		61523883-61574930		EFCAB8	EF-hand calcium binding domain 8
		61581681-61607701		SUN5	Sad1 and UNC84 domain containing 5
		61611933-61633734		BPIFB2	BPI fold containing family B member 2
		61641482-61656002		BPIFB6	BPI fold containing family B member 6
		61665357-61680683		BPIFB3	BPI fold containing family B member 3
		61689117-61711550		BPIFB4	BPI fold containing family B member 4
	38580001-38660000	38609366-38671551	Rsb*ZF_*ST*_	RIN2	Ras and Rab interactor 2
	38700001-38840000	38683625-38700031		NAA20	N(alpha)-acetyltransferase 20, NatB catalytic subunit
		38700000-38723332		CRNKL1	crooked neck pre-mRNA splicing factor 1
		38723037-38973982		CFAP61	cilia and flagella associated protein 61
14	1020001-1340000	1005889-1031106	*Rsb*	COG4	component of oligomeric golgi complex 4
		1032499-1045080		FUK	fucokinase
		1096949-1108688		ST3GAL2	ST3 beta-galactoside alpha-2,3-sialyltransferase 2
		1120895-1170694		DDX19A	DEAD-box helicase 19A
		1177773-1196812		AARS	alanyl-tRNA synthetase
		1265599-1291738		PDPR	pyruvate dehydrogenase phosphatase regulatory subunit, mitochondrial
		1316701-1359043		GLG1	golgi glycoprotein 1
16	33060001-33260000	33089170-33159243	Rsb*ZF_*ST*_	PLCXD3	16 phosphatidylinositol specific phospholipase C X domain containing 3
18	1860001- 2420000	1810732-1994082	*Rsb*	ATP10A	ATPase phospholipid transporting 10A (putative)

**Table 5:** Candidate regions and genes identified to be under selection by a combination of at least two methods in the southern Ethiopia long fat-tail vs. thin-tail sheep.

**Chr**	**Overlapping region**	**Gene location**	**Method**	**Candidate gene**	**Annotation**
2	232500001-232940000	232511525-232515117	hapFLK*ZF_*ST*_	PDE6D	phosphodiesterase 6D
		232572509-232589655		COPS7B	COP9 signalosome subunit 7B
		232749221-233048136		DIS3L2	DIS3 like 3'-5' exoribonuclease 2
	235131414-235231414	235135457-235145925	*Rsb*	FABP3	fatty acid binding protein 3
3	58380001-58700000	58404458-58464225	*Rsb*	RMND5A	required for meiotic nuclear division 5 homolog A
		58476359-58482584		D8CA	CD8a molecule
		58632941-58675138		SMYD1	SET and MYND domain containing 1
		58685544-58691134		FABP1	FABP1 fatty acid binding protein 1
	107880001-108560000	107854018-107953211		TPH2	tryptophan hydroxylase 2
		108235641-108685027		TRHDE	thyrotropin releasing hormone degrading enzyme
	181020001-181340000	181105243-181215802		SYT10	synaptotagmin 10
	220140001-220520000	220093324-220213264		ATXN10	ataxin 10
		220278340-220303444		WNT7B	Wnt family member 7B
3	106881919-107331750	107108271-107174474	*ZF_*ST*_*	TSPAN8	tetraspanin 8
3	198240001-198380000	198318924-198332818	Rsb*ZF_*ST*_	MGST1	microsomal glutathione S-transferase 1
5	46740001-47120000	46741353-46780919	hapFLK*Rsb	PKD2L2	polycystin 2 like 2, transient receptor potential cation channel
		46784304-46868016		FAM13B	family with sequence similarity 13 member B
		46910528-46915217		WNT8A	family member 8A
		46938902-46961998		NME5	NME/NM23 family member 5
		46972869-46993265		BRD8	bromodomain containing 8
		46994290-47001867		KIF20A	5 kinesin family member 20A
		47003373-47022642		CDC23	cell division cycle 23
		47062627-47080160		GFRA3	family receptor alpha 3
	47340001-47660000	47473181-47642282		CTNNA1	catenin alpha 1
		47580182-47582084		LRRTM2	leucine rich repeat transmembrane neuronal 2
		47651357-47849675		SIL1	nucleotide exchange factor
	48060001-48380000	48060550-48066158		SPATA24	spermatogenesis associated 24
		48074043-48099657		DNAJC18	DnaJ heat shock protein family
		48118011-48122197		SMIM33	small integral membrane protein 33
		48123808-48127851		TMEM173	transmembrane protein 173
		48371405-48408795		PSD2	pleckstrin and Sec7 domain containing 2
	51000001-51200000	51004236-51024531	hapFLK*ZF_*ST*_	FGF1	fibroblast growth factor 1
		51190660-51671936		ARHGAP26	Rho GTPase activating protein 26
10	29160001-29480000	28986741-29188660	*Rsb*	FRY	FRY microtubule binding protein
		29454677-29502617		RXFP2	relaxin family peptide receptor 2
11	30240001-30380000	30273281-30323323	Rsb*ZF_*ST*_	MAP2K4	mitogen-activated protein kinase kinase 4
12	69720001-69920000	69778803-69860630	hapFLK*ZF_*ST*_	LPGAT1	lysophosphatidylglycerol acyltransferase 1
		69915816-69927483		NEK2	NIMA related kinase 2
13	38520001-38660000	38609366-38671551	Rsb*ZF_*ST*_	RIN2	Ras and Rab interactor 2
18	1800001-2420000	1810732-1994082		ATP10A	ATPase phospholipid transporting 10A
19	51480001- 51680000	51478736-51488000	Rsb*ZF_*ST*_	SPINK8	serine peptidase inhibitor, Kazal type 8
		51498015-51501665		NME6	NME/NM23 nucleoside diphosphate kinase 6
		51554521-51556734		CATHL3	BAC7.5 protein
		51566237-51569729		BAC5	5 kDa bactinecin precursor
20	9480001-9740000	9408372-9492510	Rsb*ZF_*ST*_	PPARD	20 peroxisome proliferator activated receptor delta
		9523663-9535319		FANCE	Fanconi anemia complementation group E
		9551273-9570868		TEAD3	TEA domain transcription factor 3
		9574110-9588018		TULP1	tubby like protein 1
		9692040-9766541		FKBP5	FK506 binding protein 5
26	36480001-37520000	36642631-36727385	*hapFLK*	CSGALNACT1	chondroitin sulfate N-acetylgalactosaminyltransferase 1
		36739622-36795389		SH2D4A	SH2 domain containing 4A
		37045901-37438136		PSD3	pleckstrin and Sec7 domain containing 3

We performed gene ontology (GO) enrichment analysis for the candidate genes revealed in each pairwise comparison (Supplementary Table 6). The five topmost GO terms associated with the candidate genes from the E1^*^S comparison include embryonic skeletal system morphogenesis (GO:0009952, GO:0048704, GO:0030224, GO:0048706), response to cold (GO:0009409), innervation (GO:0060384), stem cell maintenance (GO:0019827) and positive regulation of cell adhesion (GO:0045785). The top GO terms associated with the E2^*^S candidate genes include cellular response to heat (GO:0034605), lipid binding (GO:0008289), magnesium ion binding (GO:0000287) and response to gamma radiation (GO:0000287). The GO terms for the genes from the E3^*^S comparison included skin development (GO:0043588), regulation of actin cytoskeleton reorganization (GO:2000249) and wound healing (GO:0042060).

## Discussion

In this study, we used Ovine 50 K SNP BeadChip generated genotype data to investigate autosomal genetic diversity in Ethiopian indigenous sheep. Including populations from other regions of the world and the African continent allowed us to assess this diversity in a global geographic context. Our findings showed that the Ethiopian indigenous sheep are genetically differentiated from the other populations including other African fat-tail sheep (Figures 2, 3). The finding that the Ethiopian fat-tail sheep are distinct from those found in North Africa, support the presence of at least two genetic groups of fat-tail sheep in the continent and two separate introduction events, one via the Northeast Africa and the Mediterranean Sea coastline, and the other via the Horn of Africa crossing through the strait of Bab-el-Mandeb, respectively. The distinct clustering of the thin-tail sheep suggests its independent introduction into the continent. The fact that the South African Ronderib and Namaqua sheep occur on the same PC planar axis with Ethiopian sheep (Figure 2) may suggest, a common genetic heritage between the two rather than with the North African breeds. The movement of sheep southwards remains speculative; some linguistic evidence suggests movement of bantu speaking populations from West Africa to South Africa through central Africa and following a western route rather than the more traditionally postulated eastern routes from East to South Africa (Newman, 1995). In such context a close clustering of the thin-tail West African sheep with some fat-tail southern African sheep breeds, such as the Namaqua from Namibia studied here is worth mentioning as it offers some possible insights. This however, will require further investigation beyond the scope of this study.

Our results agree with previous findings that were arrived at using microsatellite loci (Muigai, 2003) and 50 K SNP genotype data (Mwacharo et al., 2017). They are also in line with archaeological and anthropological evidences indicating the introduction first, of thin-tail sheep into the continent followed by fat-tail sheep, initially through the Sinai Peninsula and later the Horn of Africa region (Gifford-Gonzalez and Hanotte, 2011; Muigai and Hanotte, 2013).

Interestingly, the PCA results involving Ethiopian and Sudanese sheep separate the Ethiopian populations into three groups while ADMIXTURE revealed four genetic clusters in Ethiopian sheep irrespective of their geographic origins in the country. TreeMix revealed extensive gene flow between populations of different geographic origins and tail-types. These results suggest, most likely, current and historical intermixing of sheep following human socio-cultural and economic interactions. This appears to be a common feature in Ethiopia and most likely the Northeast and eastern Africa region as it was also observed in Ethiopian goats by Tarekegn et al. (2018). We propose here that the common D genetic background present in short fat-tail and fat-rump sheep may represent historical introgression of the thin-tail gene pool into short fat-tail and fat-rump genepool. This result calls for further investigation.

Our findings on the genetic relationships and differentiation between Ethiopian sheep populations agree with findings of previous studies, which were performed using either microsatellites (Gizaw, 2008) or 50 K SNP genotype data (Edea et al., 2017) and which indicated a grouping of Ethiopian indigenous sheep populations based on their tail phenotypes. However, uniquely in our study, the long fat-tail populations were further subdivided into two secondary groups representing sheep populations from western and southern Ethiopia (Figure 4). These two groups were also defined by different genetic backgrounds by ADMIXTURE (Figure 6) and they clustered separately in TreeMix (Figure 7). In addition, although they are defined by the same tail phenotype, the two populations of Ethiopian short fat-tail sheep did not cluster together. Geographic isolation coupled, most likely, with adaptation to different eco-climates, as well as ethnic, cultural and religious practices and differences, that can impede gene flow, may have shaped this genetic sub-structuring (Madrigal et al., 2001; Gizaw et al., 2007).

In selection signature analysis, we contrasted groups of Ethiopian indigenous sheep that showed variation in the size of the fat-tail with thin-tail sheep. Our results identified several genes as potential candidates controlling tail morphotype and fat localization in the study populations. Several genes occurred within candidate regions that overlapped between at least two of the three approaches used to detect signatures of selection *(hapFLK, F*_*ST*_*, Rsb)*. The *F*_*ST*_ approach detects signatures arising from an increase or decrease in allele frequency differentiation between populations/breeds, *hapFLK* detects the same but based on increase/decrease in haplotype frequency differentiation between populations while accounting for hierarchical population structure (Kijas, 2014) while *Rsb* detects signatures associated with the patterns of linkage disequilibrium between loci across the genome (Oleksyk et al., 2010; de Simoni Gouveia et al., 2014). Since these methods are based on different algorithms and assumptions, if common signatures are detected by at least two of the methods it suggests good reliability of the results while reducing the likelihood of interpreting false positives. They also detect signatures spanning different time periods; the *F*_*ST*_ and *hapFLK* detect signatures arising from long term differential selection while *Rsb* detects ongoing signatures of selection including those that arise in the short to medium term (Oleksyk et al., 2010).

In the E1^*^S comparison, three genes associated with growth traits were present on the candidate region on Oar2, i.e., histidine triad nucleotide binding protein 2 (*HINT2*), sperm associated antigen 8 (*SPAG8*) and natriuretic peptide receptor 2 (*NPR2*). Previous studies reported these genes to be associated with birth and carcass weights, and fat depth, respectively, in cattle (Casas et al., 2000; McClure et al., 2010) and sheep (Moradi et al., 2012; Wei et al., 2015). We also identified two genes on Oar5 (*ANGPTL8, INSR*), which might be responsible for fat accumulation in adipose tissues. Angiopoietin-like 8 (*ANGPTL8*), when induced by insulin receptor (*INSR*), inhibits lipolysis and controls post-prandial fat storage in white adipose tissue and directs fatty acids to adipose tissue for storage during the fed state (Mysore et al., 2017). The *ADAMTS3* (ADAM metallopeptidase with thrombospondin type 1 motif 3) gene was present in the region identified on Oar6. This gene is expressed in cartilage, where collagen II is a major component, as well as in embryonic bone and tendon, suggesting that it could be a major procollagen processing enzyme in musculoskeletal tissues (Dubail and Apte, 2015). The homeobox B13 (*HOXB13*) and ALX homeobox 4 (*ALX4*) were identified on the candidate region on Oar11 and Oar15, respectively. Mutations in the former result in overgrowth of caudal spinal cord and tail vertebrae in mice (Economides et al., 2003), while the latter is involved in the development of limbs and skeleton (Fariello et al., 2014).

Our enrichment analysis for the E1^*^S genes revealed a cluster of genes (*BMP4, MED1*) with functions that could possibly be related to tail formation. Bone Morphogenetic Protein 4 (*BMP4*) was revealed by *Rsb* and *F*_*ST*_ to be on a candidate region on Oar7 and it has been implicated in tail formation (Moioli et al., 2015). Peroxisome Proliferator Activated Receptor Gamma (*PPARG*) expression has been associated with back-fat thickness in sheep (Dervish et al., 2011). Ge et al. (2008) reported Mediator Complex Subunit 1 (*MED1*) is an essential protein for the optimal functioning of *PPARG*. Despite this association, our analysis did not reveal any signals spanning *PPARG*, but two of our methods (*Rsb* and *F*_*ST*_) revealed a signature on Oar20 that spanned the *PPARD* gene, a paralogue to *PPARG*.

In the same comparison (E1^*^S), we identified a cluster of genes (*CDH8, ADRB3, THRA, TRPM8, PLAC8*) that are associated with the GO biological process, response to cold. This is not surprising considering that three out of the four E1 populations are living at a high altitude and therefore in a relatively cold habitat. Indeed, Adreno receptor Beta 3 (*ADRB3*) plays a major role in energy metabolism and regulation of lipolysis and homeostasis (Wu et al., 2012). It is also associated with birth weight, growth rate, carcass composition and survival in various breeds of sheep (Horrell et al., 2009). The ion channel *TRPM8* has been reported to play a major role in eliciting cold defense thermoregulation, metabolic and defense immune responses in humans (Kozyreva and Voronova, 2015).

Several other genes occurring in the E1^*^S candidate regions and which are associated with the GO term embryonic skeletal system development (GO:0048706) included *HOXC6, SULF2, WNT11*, and *HOXB9*. *WNT11* was identified by *ZF*_*ST*_ on Oar15 while *HOXC6* and *HOXB9* were revealed by *hapFLK* on Oar3 and Oar13, respectively. The *WNT* gene family and the *T* gene have been implicated in vertebral development in laboratory mice (Greco et al., 1996), and with the short-tail phenotype in sheep (Zhi et al., 2018). In addition, the roles of the *WNT* gene family in lipid metabolic processes in fat-tail sheep have also been reported (Kang et al., 2017). The *HOX* genes represent transcriptional regulatory proteins that control axial patterning in bilaterians (Garcia-Fernàndez, 2005), where the inactivation of one of the *HOX* genes often causes transformations in the identity of vertebral elements (Mallo et al., 2010). The *HOX* genes are able to control morphologies along the anteroposterior axis (Lewis, 1978). Furthermore, *HOXC11, HOXC12*, and *HOXC13* developmental genes were found to be expressed in the tail region indicating their possible associations with tail size and fat development in fat-tail sheep (Kang et al., 2017).

The candidate regions revealed by the E2^*^S comparison, spanned 65 candidate genes. Three genes of the BPI fold Containing Family B (*BPIFB3, BPIFB4, and BPIFB6*) were present in a candidate region on Oar13. These, along with other paralogs (*BPIFB1, BPIFA3, BPIFB2, BPIFA1*), formed a cluster of functional genes related to the GO term, lipid binding functional process (Supplementary Table 6). In contrast to the E1^*^S comparison, the cluster of genes identified in the E2^*^S comparison were associated with the GO terms, Magnesium ion binding, response to gamma radiation and cellular response to heat. This suggests, most likely, the propensity of this group of sheep to adapt to the eco-climatic conditions prevailing in their home-tract. This is consistent with the humid highland and moist lowland conditions of the geographic area where the populations representing the E2 group (Bonga, Gesses, Kido) were sampled. High fecundity and prolificacy is a common reproductive trait preferred by farmers in the Bonga sheep (field observations by the last author). This may explain the occurrence of the *CIB4* and *PRKAA1* in a candidate region in the E2^*^S comparison. The *CIB4* gene was suggested to be linked, in some way, to high fecundity in the small Tail Han sheep (Yu et al., 2010) and *PRKAA1* is involved in ewe's follicular development (Foroughinia et al., 2017).

The third comparison (E3^*^S) resulted in 36 genes that occurred in candidate regions that were revealed by at least two methods used to detect selection signatures. Fatty acid binding proteins *FABP3 and FABP1* found on candidate regions on Oar2 and Oar3, respectively are the genes that relate most closely to fat deposition. *SREBF1* along with *PPARG* are the main transcription factors controlling lipogenesis in adipose tissue and skeletal muscle (Ferré and Foufelle, 2010), and are mainly regulated by fatty acid-binding proteins (*FABP*) (Lapsys et al., 2000). Recently, Bahnamiri et al. (2018) evaluated the effects of negative and positive energy balances on the expression pattern of these genes in fat-tail and thin-tail lambs. They observed differential transcriptional regulation of lipogenesis and lipolysis during periods of negative and positive energy balances in the two groups of lambs. In general, the cluster of genes identified in this comparison was significantly enriched for GO terms relating to skin development, wound healing and regulation of actin cytoskeleton reorganization (Supplementary Table 6).

The overlapped genes between all comparisons are shown in Supplementary Figure 7. The commonest genes between the three comparisons are *TSPAN8, RXFP2*, and *RIN2*. The *TSPAN8* (Tetraspanin 8) occurred in the candidate region on Oar3; it is among the genes that are reported to be associated with insulin release and sensitivity, and obesity in humans (Grarup et al., 2008), while the relaxin family peptide receptor 2 (*RXFP2*) has been associated with horn morphology (Johnston et al., 2011; Wiedemar and Drögemüller, 2015).

Twelve genes (*MELK, RNF38, GNE, CLTA, CCIN, RECK, HINT2, SPAG8, NPR2, FAM221B, MSMP, RGP1)* were common between E1^*^S and E2^*^S comparisons. On Oar2, three genes were identified within the overlapping candidate region, i.e., *CLTA* which is associated with prion protein deposition in sheep (Filali et al., 2014), *GNE* which is important for the metabolism of sialated oligosaccharides in bovine milk (Wickramasinghe et al., 2011) and *RECK* which encodes an inhibitor of angiogenesis, invasion and metastasis, DNA methylation, and increased mRNA in cell lines in humans (Su, 2012). Other genes (i.e., *HINT2, SPAG8*, and *NPR2*) are associated with fat deposition in sheep as herein discussed for each of the three comparisons.

Furthermore, one gene (*DIS3L2*) was in a candidate region that overlapped between the E1^*^S and E3^*^S comparisons. DIS3 like 3'-5' exoribonuclease 2 (*DIS3L2*) has also been identified, among genes involved in cancer, cellular function and maintenance, and neurological disease, in a candidate region under selection in cattle (Gautier et al., 2009). In sheep, using *F*_*ST*_, *iHS*, and *Rsb*, de Simoni Gouveia et al. (2017) indicated that *DIS3L2* is among genes associated with height variation. In addition, *DIS3L2* has reportedly been associated with the Perlman syndrome, which is characterized by overweight in humans (Astuti et al., 2012).

Finally, seventeen genes (*PKD2L2, FAM13B, WNT8A, NME5, BRD8, KIF20A, CDC23, GFRA3, CTNNA1, LRRTM2, SIL1, SPATA24, DNAJC18, SMIM33, TMEM173, FRY, ATP10A)* were in candidate regions that overlapped between the E2^*^S and E3^*^S comparisons. Among these, DnaJ heat shock protein family (*HSP40*) member C18 (*DNAJC18*) and spermatogenesis associated 24 (*SPATA24*) on Oar5 were reported among genes involved in heat stress tolerance and male reproductive function, respectively, in East African Shorthorn Zebu cattle (Bahbahani et al., 2015).

## Conclusion

Overall, our results revealed four distinct autosomal genomic backgrounds (A, B, C, D) in Ethiopian indigenous sheep. The genotypes of most of the individuals analyzed were made up of at least two genetic backgrounds which could be accounted for by some level of current or historical admixture between populations. Selection signature analysis identified several putative candidate regions spanning genes relating to skeletal structure and morphology, fat deposition and possibly adaptation to environmental selection pressures. Our results indicate that Ethiopian indigenous sheep could be a valuable animal genetic resource that can be used to understand genetic mechanisms associated with body fat metabolism and distribution. This is especially important because fat deposits are a crucial component of adaptive physiology and excessive fat deposition in adipose tissue can result in obesity and overweight, and energy metabolism disorders in humans.

## Data Accessibility

Genotypic data of 160 animals representing eleven Ethiopian and two Sudanese sheep populations are deposited and available at (https://www.animalgenome.org/repository/pub/NOTT2018.0423/).

## Ethics Statement

The animals used in this study are owned by farmers. Prior to sampling, the objectives of the study were explained to them in their local languages so that they could make an informed decision regarding giving consent to sample their animals. Government veterinary, animal welfare and health regulations were observed during sampling. The procedures involving sample collection followed the recommendation of directive 2010/63/EU. Skin tissues importation and/or exportation was permitted by the Ethiopian Ministry of Livestock and Fisheries under Certificate No: 14-160-401-16.

## Author Contributions

AbA, JM, and OH conceived and designed the study. AbA analyzed the data and together with JM wrote the manuscript. JM and OH revised the manuscript. HB provided support in data analysis. SM, FP, and EC contributed to genotyping and genotype data of non-Ethiopian breeds (Najdi, Omani, and Libyan Barbary) and provided critical inputs on data analysis and in writing the manuscript. FA, MA, and MOA supported the sampling and genotyping of Najdi, Omani and Libyan sheep. AK and AyA lead and coordinated the sampling of Ethiopian sheep HM lead and coordinate the sampling of Sudanese sheep. All authors contributed to the interpretation of the results based on their knowledge on local indigenous sheep genetic resources of their respective countries. All the authors read and approved the final manuscript.

### Conflict of Interest Statement

The authors declare that the research was conducted in the absence of any commercial or financial relationships that could be construed as a potential conflict of interest.

## Supplementary Material

Supplementary Figure 1 Patterns of linkage disequilibrium (LD) calculated within the Ethiopian (ET) and Sudanese (SD) sheep populations.

Supplementary Figure 2 Distribution of the standardized *Z*-score values for **(A)** *hapFLK* and **(B)** *F*_*ST*_ for the autosomal markers.

Supplementary Figure 3 Distribution of genetic diversity indices within each breed. **(A)** SNP displaying polymorphism (*Pn)*, **(B)** Expected heterozygosity *(He*); Observed heterozygosity (*Ho*); **(C)** Inbreeding coefficient (*F*).

Supplementary Figure 4 Genetic variation among the Ethiopian sheep populations in a global geographic context (all animals included for each population).

Supplementary Figure 5 Distribution of genetic variation among the worldwide fat-tail sheep (all animals included for each population).

Supplementary Figure 6 Cross-validation error plot for admixture analysis of the studied populations **(A)** in the national and **(B)** in the global context.

Supplementary Figure 7 Venn diagram showing the distribution and number of genes shared between the three groups of sheep (E1, E2, E3, S) used in the analysis of selection signatures.

Supplementary Table 1 Description of the world-wide breeds of sheep used in the study.

Supplementary Table 2 Proportion of the genetic backgrounds in each study population as identified by Admixture analysis.

Supplementary Table 3 Results of *f4* statistics for the study breeds as generated with TreeMix.

Supplementary Table 4 Results of *f3* statistics for the study breeds as generated with TreeMix.

Supplementary Table 5 Results of signature of selection between the three population groups.

Supplementary Table 6 Enriched functional term clusters and their enrichment scores following *DAVID* analysis for genes identified in the candidate regions under selection.
