# Supplementary figures and images for: Genome-Wide Variation, Candidate Regions and Genes Associated With Fat Deposition and Tail Morphology in Ethiopian Indigenous Sheep
Source: Front Genet. 2019 Jan 9;9:699. doi: 10.3389/fgene.2018.00699 (PMC6334744; doi:10.3389/fgene.2018.00699)

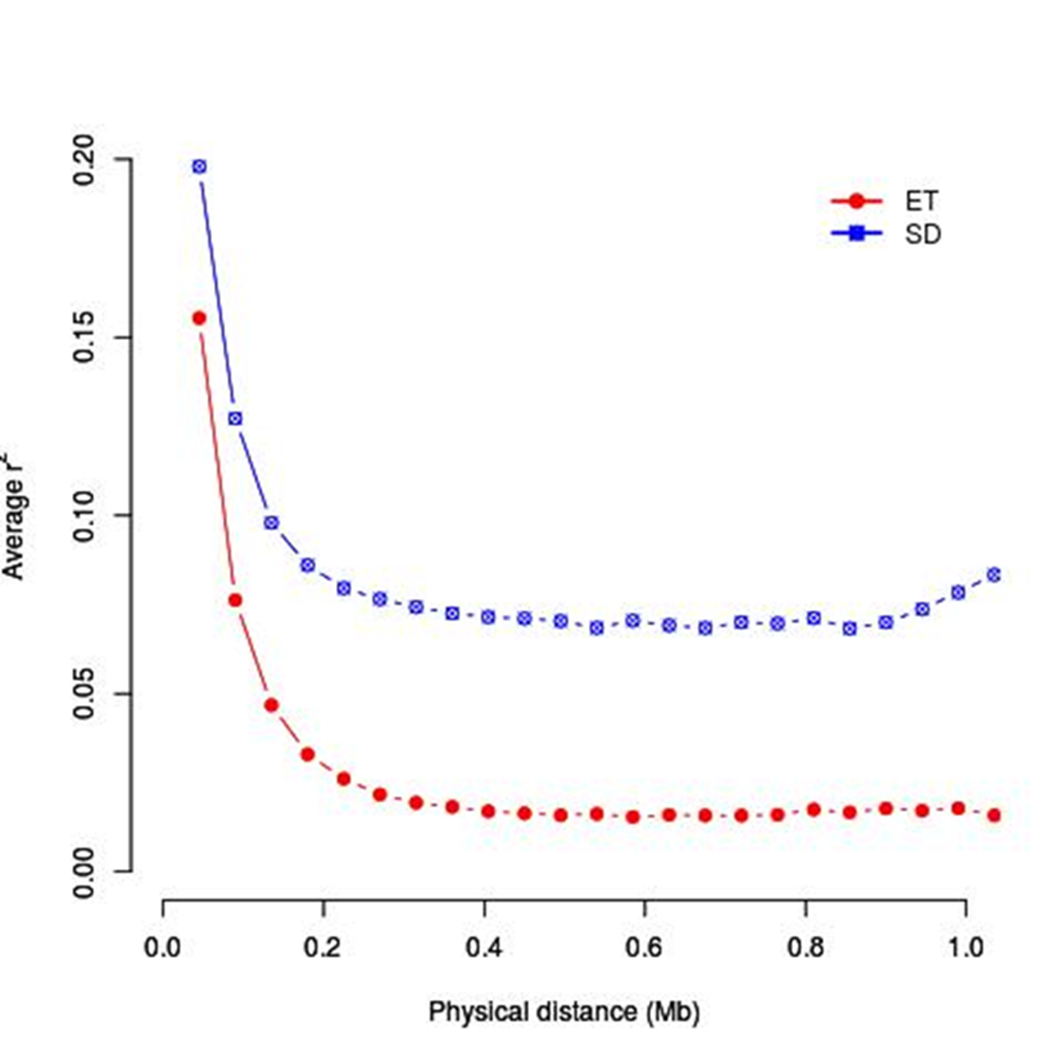


**Supplementary Figure S1**

Supplement: Supplementary Figure 1 — Patterns of linkage disequilibrium (LD) calculated within the Ethiopian (ET) and Sudanese (SD) sheep populations. [file Data_Sheet_1.docx]

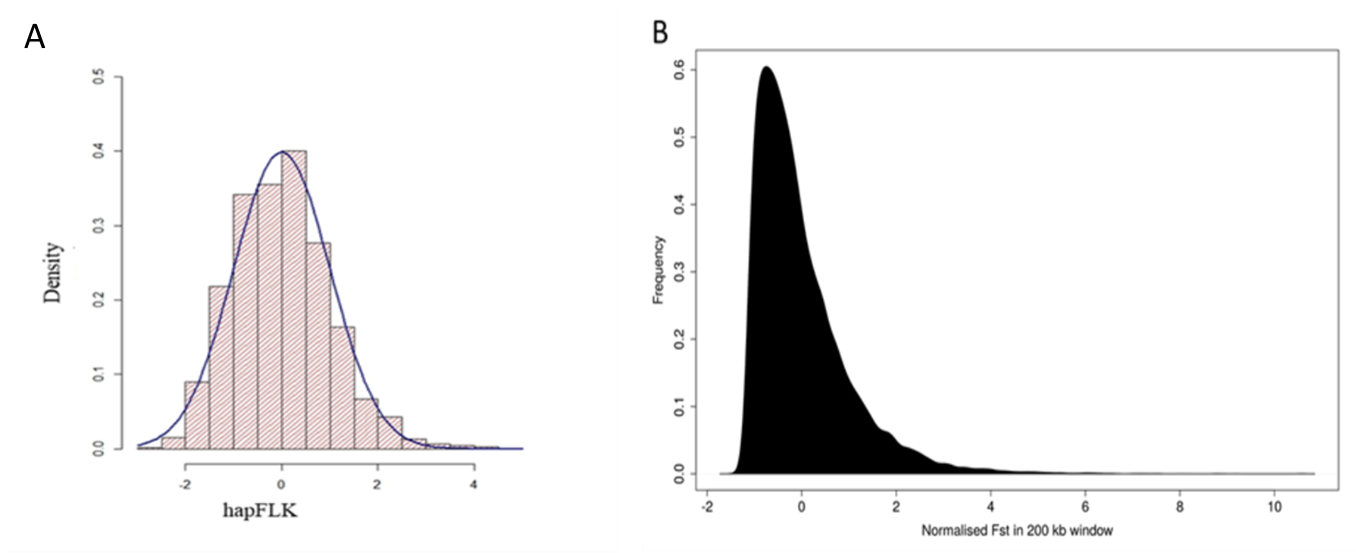


**Supplementary Figure S2**

Supplement: Supplementary Figure 2 — Distribution of the standardized Z-score values for (A) hapFLK and (B) FST for the autosomal markers. [file Data_Sheet_2.docx]

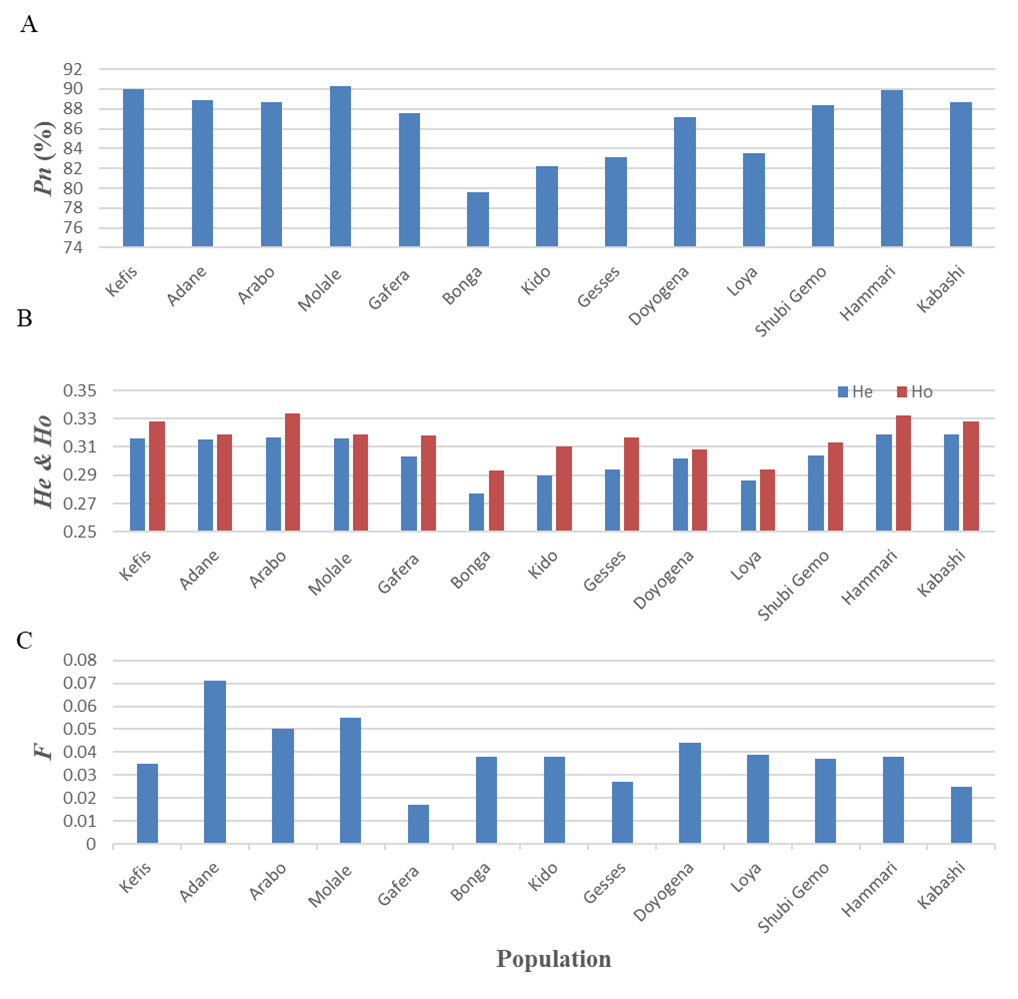


**Supplementary Figure S3**

Supplement: Supplementary Figure 3 — Distribution of genetic diversity indices within each breed. (A) SNP displaying polymorphism (Pn), (B) Expected heterozygosity (He); Observed heterozygosity (Ho); (C) Inbreeding coefficient (F). [file Data_Sheet_3.docx]

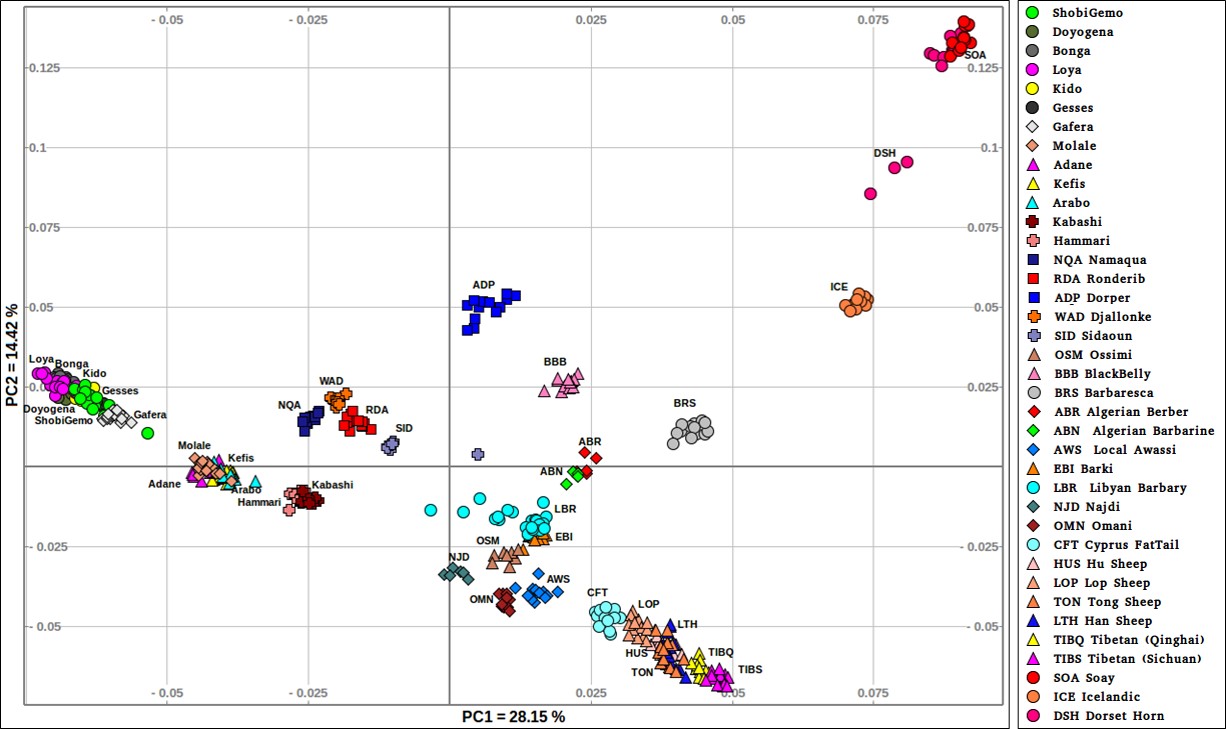


**Supplementary Figure S4**

Supplement: Supplementary Figure 4 — Genetic variation among the Ethiopian sheep populations in a global geographic context (all animals included for each population). [file Data_Sheet_4.docx]

***
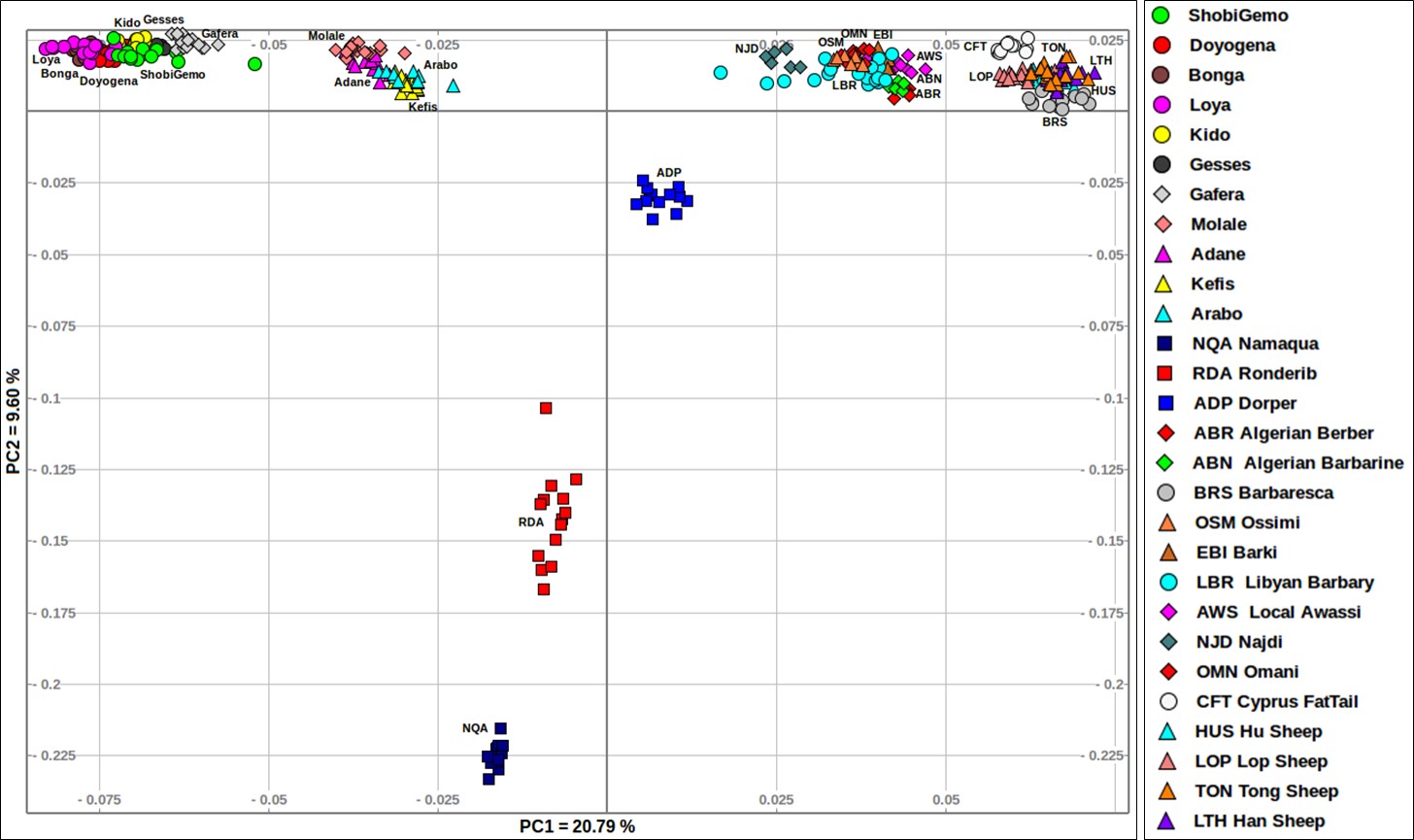
***

**Supplementary Figure S5**

Supplement: Supplementary Figure 5 — Distribution of genetic variation among the worldwide fat-tail sheep (all animals included for each population). [file Data_Sheet_5.docx]

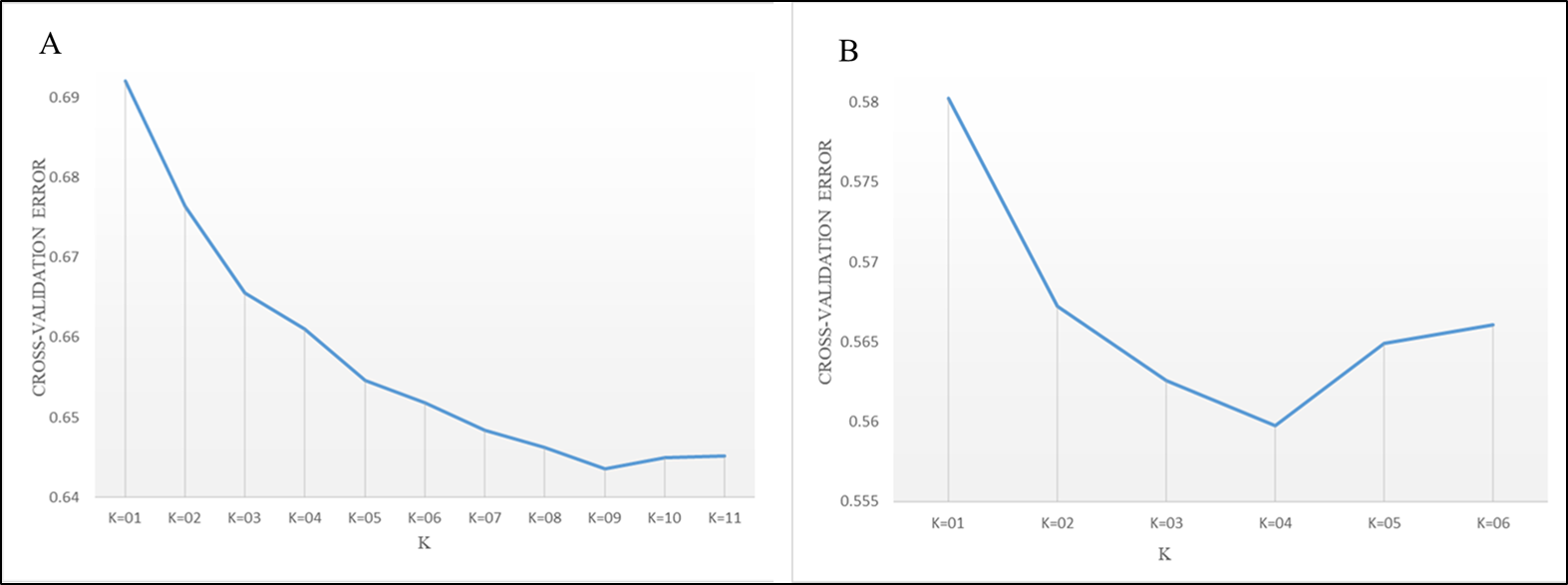


**Supplementary Figure S6**

Supplement: Supplementary Figure 6 — Cross-validation error plot for admixture analysis of the studied populations (A) in the national and (B) in the global context. [file Data_Sheet_6.docx]

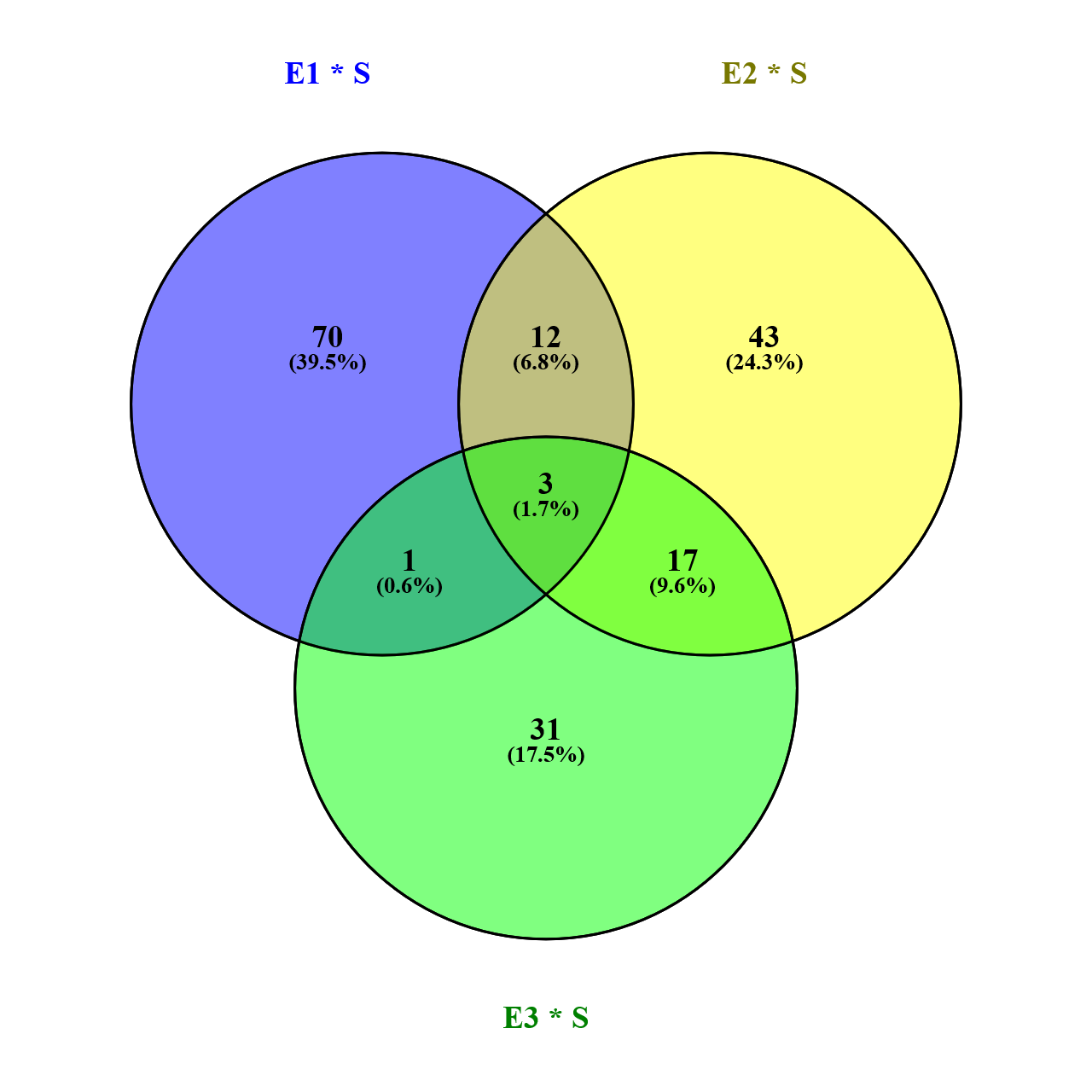


**Supplementary Figure S7**

Supplement: Supplementary Figure 7 — Venn diagram showing the distribution and number of genes shared between the three groups of sheep (E1, E2, E3, S) used in the analysis of selection signatures. [file Data_Sheet_7.docx]
