# Supplementary material for: Genome-Wide Variation, Candidate Regions and Genes Associated With Fat Deposition and Tail Morphology in Ethiopian Indigenous Sheep
Source: Front Genet. 2019 Jan 9;9:699. doi: 10.3389/fgene.2018.00699 (PMC6334744; doi:10.3389/fgene.2018.00699)
Supplement: Supplementary Table 1 — Description of the world-wide breeds of sheep used in the study. [file Table_1.docx]

**Supplementary Table S1** Description of the world wide breeds used in the study

| **Country of origin** | **Breed** | **N** | **Abbr.** | **Tail type** | **Coat type** | **Source** |
| --- | --- | --- | --- | --- | --- | --- |
| Italy | Barbaresca | 13 | BRS | Fat-tailed | Wool | Mastrangelo et al., 2017 |
| Libya | Libyan Barbary | 24 | LBR | Fat-tailed | Coarse-wooled | Mastrangelo et al., 2018 |
| Algeria | Berber | 6 | ABR | Fat-tailed | Wool | Gaouar et al., 2017 |
|  | Sidaoun | 6 | SID | Long-tailed | Hair |  |
|  | Barbarine | 5 | ABN | Fat-tailed | Coarse-wooled |  |
| Egypt | Ossimi | 8 | OSM | Fat-tailed | Coarse-wooled | Mastrangelo et al., 2018 |
|  | Barki | 13 | EBI | Fat-tailed | Coarse-wooled | Kijas et al., 2012 |
| Israel | Local Awassi | 13 | AWS | Fat-tailed | Coarse-wooled |  |
| UK | Dorset Horn | 13 | DSH | Thin-tailed | Fine wool |  |
| UK | Soay | 12 | SOA | Short tail | Wool |  |
| Iceland | Icelandic | 13 | ICE | Fluke-shaped tail | Fine wool |  |
| Cyprus | Cyprus FatTail | 14 | CYFT | Fat-tailed | Coarse-wooled |  |
| South Africa | African Dorper | 13 | ADP | Short fat-tail | Coarse-wooled |  |
|  | Namaqua Afrikaner | 13 | NQA | Long fat-tailed | Coarse-wooled |  |
|  | Ronderib Afrikaner | 13 | RDA | Long fat-tailed | Coarse-wooled |  |
| Caribbean island | Barbados BlackBelly | 13 | BBB | Thin-tailed | Hair |  |
| China | Hu sheep | 12 | HUS | Short fat-tailed | Wool | Yuan et al., 2017 |
|  | Tong sheep | 15 | TON | Long fat-tailed | Wool |  |
|  | Han sheep | 15 | LTH | Large-tailed | Wool |  |
|  | Lop sheep | 13 | LOP | Short fat-tailed | Wool |  |
|  | Tibetan (From Qinghai) | 14 | TIBQ | Thin-tailed | Wool |  |
|  | Tibetan (From Sichuan) | 14 | TIBS | Thin-tailed | Wool |  |
| West Africa | Djallonke | 10 | WAD | Thin-tailed | Hair | Spangler et al., 2017 |
| Saudi Arabia | Najdi | 6 | NJD | Fat-tailed | Coarse-wooled | Faisal Almathen and Fabio Pilla |
| Oman | Omani | 10 | OMN | Fat-tailed | Coarse-wooled | Mohammed Al Abri and Fabio Pilla |
| **Total** | | **301** |  | | | |

Gaouar, S.B.S., Lafri, M., Djaout, A., El-Bouyahiaoui, R., Bouri, A., Bouchatal, A., Maftah, A., Ciani, E. and Da Silva, A.B., (2017). Genome-wide analysis highlights genetic dilution in Algerian sheep. *Heredity*, 118(3), p.293.

Kijas, J.W., Lenstra, J.A., Hayes, B., Boitard, S., Neto, L.R.P., San Cristobal, M., Servin, B., McCulloch, R., Whan, V., Gietzen, K. and Paiva, S., 2012. Genome-wide analysis of the world's sheep breeds reveals high levels of historic mixture and strong recent selection. *PLoS biology*, *10*(2), p.e1001258.

Mastrangelo, S., Moioli, B., Ahbara, A., Latairish, S., Portolano, B., Pilla, F.,Ciani, E. (2018). A genome-wide scan of fat-tail sheep identifies signals of selection for fat deposition and adaptation. *Anim Prod Sci*. (<http://www.publish.csiro.au/AN/justaccepted/AN17753>)

Mastrangelo, S., Portolano, B., Di Gerlando, R., Ciampolini, R., Tolone, M., Sardina, M. T. (2017). Genome-wide analysis in endangered populations: a case study in Barbaresca sheep. *Animal*. 11, 1107-1116.

Spangler, G.L., Rosen, B.D., Ilori, M.B., Hanotte, O., Kim, E.S., Sonstegard, T.S., Burke, J.M., Morgan, J.L., Notter, D.R. and Van Tassell, C.P. (2017). Whole genome structural analysis of Caribbean hair sheep reveals quantitative link to West African ancestry. *PloS One*, *12*(6), p.e0179021

Yuan, Z., Liu, E., Liu, Z., Kijas, J.W., Zhu, C., Hu, S., et al. (2017). Selection signature analysis reveals genes associated with tail type in Chinese indigenous sheep. *Anim. Genet*. 48(1), 55-66.
