# Supplementary material for: Genome-Wide Variation, Candidate Regions and Genes Associated With Fat Deposition and Tail Morphology in Ethiopian Indigenous Sheep
Source: Front Genet. 2019 Jan 9;9:699. doi: 10.3389/fgene.2018.00699 (PMC6334744; doi:10.3389/fgene.2018.00699)
Supplement: Supplementary Table 2 — Proportion of the genetic backgrounds in each study population as identified by Admixture analysis. [file Table_2.docx]

**Supplementary Table S2** Proportions of the genetic admixture backgrounds in each population

| **Breed** | **Proportions of the genetic backgrounds (%)** | | | |
| --- | --- | --- | --- | --- |
|  | **A** | **B** | **C** | **D** |
| Kefis | - | - | 30 | 70 |
| Arabo | - | - | 40 | 60 |
| Adane | - | 5 | 85 | 10 |
| Molale (Menz) | - | - | 80 | 20 |
| Gafera (Washera) | - | 60 | 40 | - |
| Bonga | 10 | 90 | - | - |
| Kido | 5 | 90 | 5 | - |
| Gesses | 2 | 93 | 2 | 3 |
| Doyogena | 50 | 40 | 10 | - |
| Loya | 90 | 10 | - | - |
| ShubiGemo | 65 | 10 | 20 | 5 |
| Hammari | - | 10 | - | 90 |
| Kabashi | - | 10 | - | 90 |
