# Supplementary material for: Genome-Wide Variation, Candidate Regions and Genes Associated With Fat Deposition and Tail Morphology in Ethiopian Indigenous Sheep
Source: Front Genet. 2019 Jan 9;9:699. doi: 10.3389/fgene.2018.00699 (PMC6334744; doi:10.3389/fgene.2018.00699)
Supplement: Supplementary Table 6 — Enriched functional term clusters and their enrichment scores following DAVID analysis for genes identified in the candidate regions under selection. [file Table_6.docx]

**Supplementary Table S6** Enriched functional term clusters and their enrichment scores following *DAVID* analysis for genes identified in Ethiopian and Sudanese sheep

| **ID** | **Term** | **P value** | **Associated genes** | **Comparison** |
| --- | --- | --- | --- | --- |
| GO:0009952 | anterior/posterior pattern specification | 0.0001 | *HOXB3, HOXC6, HOXB4, HOXB1, HOXC8, HOXB2, HOXB7, HOXB5, HOXC4, HOXB6, HOXB9* | *Fat-rump vs Thin-tail* |
| GO:0048704 | embryonic skeletal system morphogenesis | 0.0010 | *HOXB3, HOXB4, HOXB1, HOXB2, HOXB7, HOXB5, HOXB6* | *Fat-rump vs Thin-tail* |
| GO:0009409 | response to cold | 0.0040 | *CDH8, ADRB3, THRA, TRPM8, PLAC8* | *Fat-rump vs This-tail* |
| GO:0030224 | monocyte differentiation | 0.0045 | *BMP4, MED1* | *Fat-rump vs Thin-tail* |
| GO:0048706 | embryonic skeletal system development | 0.0096 | *HOXC6 SULF2, WNT11, HOXB9* | *Fat-rump vs thin-tail* |
| GO:0060384 | Innervation | 0.0149 | *SULF2, RNF165, LRIG2, UNC13B* | *Fat-rump vs thin-tail* |
| GO:0019827 | stem cell population maintenance | 0.0161 | *MED28, NODAL, DIS3L2, MED24, LEO1, FZD7* | *Fat-rump vs thin-tail* |
| GO:0045785 | positive regulation of cell adhesion | 0.0209 | *VAV3, ERBB2, ITGAV, ANGPT1, SKAP1* | *Fat-rump vs thin-tail* |
| GO:0034605 | cellular response to heat | 0.0033 | *TFEC, CLPB, NF1, SLC52A3, MYOF* | *Western long fat-tail vs thin-tail* |
| GO:0008289 | lipid binding | 0.0051 | *BPIFB1, BPIFA3, BPIFB2, BPIFB3, BPIFB4, BPIFA1, BPIFB6* | *Western long fat-tail vs thin-tail* |
| GO:0000287 | magnesium ion binding | 0.0069 | *GSS, CIB4, EYA2, GTPBP10, SNCA, ATP10A, DIS3L2, ERN1, ITPK1, STK3, ADPRH* | *Western long fat-tail vs thin-tail* |
| GO:0010332 | response to gamma radiation | 0.0162 | *BRCA2, TRIM13, PRKDC, PRKAA1* | *Western long fat-tail vs thin-tail* |
| GO:0043588 | skin development | 0.0027 | *COL3A1, ITGA3, PTCH2, ARRDC3, COL5A2, DHCR24* | *Southern long fat-tail vs thin-tail* |
| GO:2000249 | regulation of actin cytoskeleton reorganization | 0.0072 | *GMFG, SEMA3E, RAPGEF3, ARHGDIB* | *Southern long fat-tail vs thin-tail* |
| GO:0042060 | wound healing | 0.0232 | *PPARD, COL3A1, NF1, GRHL3, PAK1* | *Southern long fat-tail vs thin-tail* |
